# Piecing Together Large Polycyclic Aromatic Hydrocarbons and Fullerenes: A Combined ChemTEM Imaging and MALDI-ToF Mass Spectrometry Approach

**DOI:** 10.3389/fchem.2021.700562

**Published:** 2021-06-14

**Authors:** R. K. E. Gover, T. W. Chamberlain, P. J. Sarre, A. N. Khlobystov

**Affiliations:** ^1^ School of Chemistry, The University of Nottingham, Nottingham, United Kingdom; ^2^ Institute of Process Research and Development, School of Chemistry, University of Leeds, Leeds, United Kingdom

**Keywords:** PAH, fullerene, chemTEM, self-assembly, bottom-up

## Abstract

Motivated by their importance in chemistry, physics, astronomy and materials science, we investigate routes to the formation of large polycyclic aromatic hydrocarbon (PAH) molecules and the fullerene C_60_ from specific smaller PAH building blocks. The behaviour of selected PAH molecules under electron (using transmission electron microscopy, TEM) and laser irradiation is examined, where four specific PAHs—anthracene, pyrene, perylene and coronene—are assembling into larger structures and fullerenes. This contrasts with earlier TEM studies in which large graphene flakes were shown to transform into fullerenes *via* a top-down route. A new combined approach is presented in which spectrometric and microscopic experimental techniques exploit the stabilisation of adsorbed molecules through supramolecular interactions with a graphene substrate and enable the molecules to be characterised and irradiated sequentially. Thereby allowing initiation of transformation and characterisation of the resultant species by both mass spectrometry and direct-space imaging. We investigate the types of large PAH molecule that can form from smaller PAHs, and discuss the potential of a “bottom-up” followed by “top-down” mechanism for forming C_60_.

## Introduction

There is widespread interest in planar aromatic and fullerenic carbon macromolecules, and in transformations between these molecular forms. The significance of these structures extends across organic, environmental, combustion, biomedical and materials chemistry, and, particularly in the context of this paper, carbon quantum dots (Tian et al., 2018; Yan et al., 2019; Semeniuk et al., 2019), and the chemistry and physics of these molecules in astronomy (Tielens, 2013). Polycyclic aromatic hydrocarbon (PAH) molecules were first suggested to be of importance in an astronomical context by Donn (1968) and are now known to be present in numerous astrophysical environments (Tielens, 2008; Tielens, 2013). The C_60_ molecule, discovered serendipitously in experiments motivated by astronomical questions (Kroto et al., 1985), has been found in a wide range of astronomical sources from planetary nebulae to young stellar objects and the interstellar medium (see references in Roberts et al., 2012; Berné et al., 2015a; Berné et al., 2015b; Maier and Campbell, 2016; Linnartz et al., 2020).

In this paper we report electron- and photon-promoted self-assembly experiments in which small planar aromatic molecules ranging in size from anthracene (C_14_H_10_) to coronene (C_24_H_12_), adsorbed on a graphitic surface, are exposed to an electron beam or laser radiation, in which chemical evolution to form larger PAHs and C_60_ is probed by transmission electron microscopy and mass spectrometry. We first briefly review “top-down” and “bottom-up” supramolecular pathways to carbon macromolecule formation.

In respect of “top-down” formation, high-resolution TEM has revealed that nm-sized flakes of graphene can transform into C_60_ as a result of high energy electron beam (e-beam) irradiation (Chuvilin et al., 2010). This occurs through loss of carbon atoms from the graphene edge, resulting in the formation of pentagons which is considered to be an essential step in folding the planar structure to form a fullerene cage. The mechanism proposed included calculated energies of the required intermediates and revealed stability of the fullerene structure to be the overall driving force in the formation process (Chuvilin et al., 2010). Comparison between the TEM results and conditions in the interstellar medium led to a proposed “top-down” C_60_ formation mechanism involving UV photo-processing of large PAH molecules, which are known to be ubiquitous in space (Berné and Tielens, 2012; Tielens, 2008). The “top-down” mechanism in this case involves UV-photolysis-initiated dehydrogenation of PAHs containing approximately 70 carbon atoms to form small graphene flakes (Goroff, 1996), loss of a carbon atom, followed by pentagon formation within the structure. This has been explored theoretically in a study carried out to calculate IR spectra of intermediates in the fullerene formation process to make comparison with IR interstellar emission features (Galué, 2014). This top-down model is supported by laboratory studies that have revealed photofragmentation patterns of large PAHs (>60C atoms) which are consistent with the formation of C_60_ (Zhen et al., 2014). Recent modelling of the conversion of circumovalene (C_66_H_20_) to C_60_ suggests that only PAH molecules with between 60 and 66 carbon atoms may be of practical significance for the formation of C_60_ in the NGC 7023 nebula due to the extended timescale required for loss of C_2_ units in the shrinking of larger structures (Berné et al., 2015a).

The “bottom-up” formation of large PAHs/quantum dots and C_60_ from smaller molecular precursors has received increasing attention recently from both terrestrial and astronomical perspectives through both gas and surface reactions. For generation of large PAHs on non-metallic surfaces, approaches include thermal fusion of coronene (Talyzin et al., 2011) and pentacene (Ishii et al., 2011), soft X-ray irradiation of pentacene (Heya et al., 2020), and oligomerization of dehydrogenated PAHs deposited as cations from the gas phase (Weippert et al., 2020). In respect of fullerene formation, this route is supported by experimental observation of C_60_ formation during the pyrolysis of PAHs (Taylor et al., 1993; Osterodt et al., 1996), laser pyrolysis of hydrocarbons (Ehbrecht et al., 1993; Armand et al., 1997) and laser desorption ionisation (LDI) of triphenylene in an experiment that combines LDI with ion-mobility mass spectrometry (Han et al., 2016). Gas-phase formation of large PAHs includes conversion of ionic pyrene clusters under laser irradiation (Zhen et al., 2018), and pyrene-dicoronylene and hexabenzocoronene-anthracene equivalents (Zhen 2019; Zhen et al., 2019). Separately, isolable quantities of C_60_ have been synthesized in 12 steps from commercially available starting materials, finally proceeding via a molecular polycyclic aromatic chlorine substituents at key positions which forms C_60_ when subjected to flash vacuum pyrolysis at 1,100°C (Scott et al., 2002).

In this work we investigate the fate of small PAHs under experimental laboratory conditions in which C-H bonds are broken photochemically or by electron impact; ionisation of the samples may also occur. Our approach builds on the previously reported ChemTEM methodology of encapsulating molecules in nanosized carbon test tubes and simultaneously initiating and interrogating molecular reactions using a beam of electrons as a local-probe/local-stimulus simultaneously (Skowron et al., 2017). Herein, we explore a low-dimensional material, graphene sheets, as a nano-sized petri dish, to connect PAH molecules with the macroworld, i.e., hold them in place on the TEM grid (Markevich et al., 2015; Mirzayev et al., 2017), and utilise both local (e-beam) and macro-probes (a beam of photons) to investigate the chemistry of PAHs. PAHs have affinity for graphene (Björk et al., 2010; Dappe et al., 2015) and readily form stable supramolecular complexes (e.g., PAHs adsorbed on graphene by π - π interactions), which allows us to study the molecules both by TEM and by matrix-assisted laser desorption/ionisation time-of-flight mass spectrometry (MALDI-TOF MS). This combined approach enables the correlation of data from the local-probe and bulk-probe analysis methods for the first time, and reveals important aspects of the molecular polycondensation process, including intermediate species, and determine mechanistic details linking specific molecular precursors with formation of large PAHs and C_60_. In addition, our new methodology was designed and implemented to utilise both of the aforementioned experimental techniques to enable characterisation of species and initiation of reactions within an identical location of a sample, so that transformation processes and molecular speciation could be carried out at each step of the experiment.

## Results and Discussion

### Electron-Beam Irradiation of PAHs

TEM is a useful tool for probing transformations of carbon species with a vacuum chamber pressure of approx. 10^−12^  mbar and high-energy electrons to initiate transformations. It has been shown by Chuvilin et al. that under these conditions graphene flakes can undergo transformations that result in the formation of fullerene molecules (Chuvilin et al., 2010). However, in our study irradiation by the electron beam of TEM was carried out on discrete PAH molecules, rather than non-uniform flakes of graphene, in order to initiate and monitor transformations of these molecules, and hence to elucidate the processes, including fullerene formation, that occur. In these experiments, the electrons can transfer sufficient energy to remove hydrogen atoms from PAHs (Chamberlain et al., 2015) and promote rearrangement of carbon atoms, while imaging these induced transformations as they are occurring.

**FIGURE 1:**
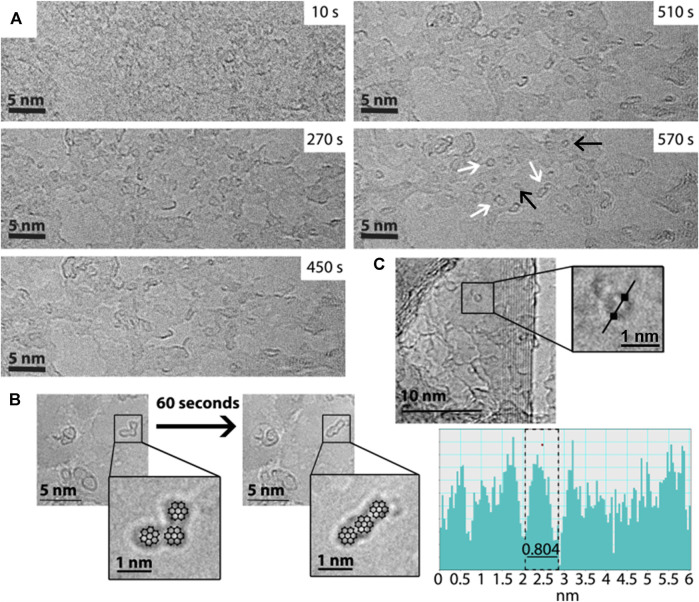
**(A)** HRTEM time series of images showing the transformation from a spatially undefined starting sample of perylene (at 10 s) to well-defined “islands” or oligomers (570 s) and fullerene structures adsorbed on surface of graphite support. White arrows indicate examples of newly formed, stable oligomers and black arrows show fullerene structures. **(B)** HRTEM images showing the evolution of PAH oligomers, resulting from the irradiation of coronene over a time period of ∼60 s. As a guide; coronene structures are overlaid the micrographs in the zoomed-in inserts. **(C)** HRTEM showing an example of fullerene structure (see zoomed-in region), formed as a result of prolonged irradiation of coronene, alongside a line profile plot showing the structure to have a diameter comparable to C_60_ (0.8 ± 0.1 nm).

A full description of all experimental procedures and materials is provided in the Supplementary Material. TEM was carried out on the PAH molecules anthracene, pyrene, perylene and coronene. These molecules were supported on few-layers graphene—to dissipate any ionisation and heating effects caused by the e-beam—and then irradiated by the e-beam in TEM for approximately 30 min, with images taken periodically at intervals of 1.5 min (on average). Under these conditions, chemical transformations are driven by the momentum of the fast electrons which is transferred directly to the atoms within the molecules (Supplementary Material). All experiments were performed using a 100 keV e-beam and a dose rate of approximately 10^9^ e^−^ nm^−2^ s^−1^, under which conditions the knock-on damage experienced by the PAH molecules leads predominantly to ejection of their hydrogen atoms (Chamberlain et al., 2015). The PAH molecules are left with very reactive carbon radical sites at their edges; these react quickly via dimerisation or cross-coupling reactions, to create new C-C bonds, culminating in the formation of PAH “oligomers.” These can be thought of either as larger PAH molecules, or as small graphene-like flakes (sp^2^ islands). The formation of oligomeric species is seen to occur for all four PAH samples (see Supplementary Material for a complete set of time series images for each PAH, and videos), with the example of perylene being given in Figure 1A. In this figure, the initial frame of the time series shows no defined structures, whereas in the final frame well-defined shapes with dark, high-contrast edges can be seen, indicating the formation of discrete new molecular species on the carbon surface. These species increase in size over the duration of the experiment (Supplementary Table S1), suggesting addition to, rather than fragmentation of, the original molecule; hence, this supports the idea of growth and formation of PAH aggregates and “oligomers.” The influence of the e-beam on the molecules and oligomers is seen due to the changes in the shapes of the structures that are observed. This is demonstrated in Figure 1B, which shows the example of coronene under the e-beam, and the change in shape of one of these structures over time. For illustrative purposes the structures of a number of coronene molecules are depicted over the TEM micrograph; in this way shapes seen in the micrograph can more easily be visualised as individual molecules or oligomers. After prolonged e-beam irradiation examples are seen in which the more circular PAH oligomers appear to have “rolled” up to form fullerene-type structures, in a manner similar to the process described by Chuvilin et al. (2010). We propose that under the conditions of our experiments the structure of the PAH oligomer is crucial as to whether it can be transformed into C_60_ over time, for example conversion of the collinear coronene species shown in Figure 1B to C_60_ would involve a large number of C-C bond breaking events and thus is unlikely to occur (see Supplementary Section S6 for full details). Figure 1C shows an example of a fullerene structure formed during the e-beam exposure of coronene. This has a measured diameter of ∼0.8 nm, which is comparable to the diameter of C_60_ (c.f. the crystallographic diameter of C_60_ = 0.71 nm, see Liu et al., 1991). Similar fullerene structures are also observed for perylene, as highlighted in Figure 1A, Supplementary Figure S3. It is important to note that irradiation of control samples in which blank graphite substrates were present showed no evidence of the formation of PAH oligomers or C_60_ (see Supplementary Material for details).

The PAH oligomers formed as a result of these transformations are found to be stable, as demonstrated by their long lifetime under the e-beam. There are a number of published examples in which PAH molecules are seen to polymerize in this way under the electron beam in TEM experiments (Talyzin et al., 2011; Fujihara et al., 2012; Botka et al., 2014). For example, the commonly seen transformation of coronene to dicoronylene can happen on the coming together of two coronene molecules, having ejected one or more hydrogen atoms, with the driving force being the formation of a new stable, six-membered ring between the two, and the overall conservation of planarity and aromaticity in the final molecule. In this way, it can be seen how bottom-up construction of larger PAHs, or PAH oligomers, can be a facile process under these irradiation conditions.

### UV Irradiation of PAHs

Many of the transformations likely to occur in the ISM are initiated by the irradiation of molecules with UV photons or high-energy cosmic rays. Hence, the photo-processing of PAHs, with a focus on fullerene formation, was investigated using a source of UV photons. MALDI-TOF MS is traditionally used to characterize molecules and identify molecular structures and causes little damage to the sample as it uses low UV laser fluence to vaporize and ionise the molecular species. However, if the fluence of the UV laser is increased, it can be used as a source of UV photons which are capable of initiating transformations within a sample. UV irradiation of pyrene was carried out for a range of values of laser fluence; at low laser fluence the mass peak for pyrene (m/z = 202) dominates, whereas at higher fluence heavier molecules form and are detected (for mass spectra see Supplementary Figure S5).

**FIGURE 2:**
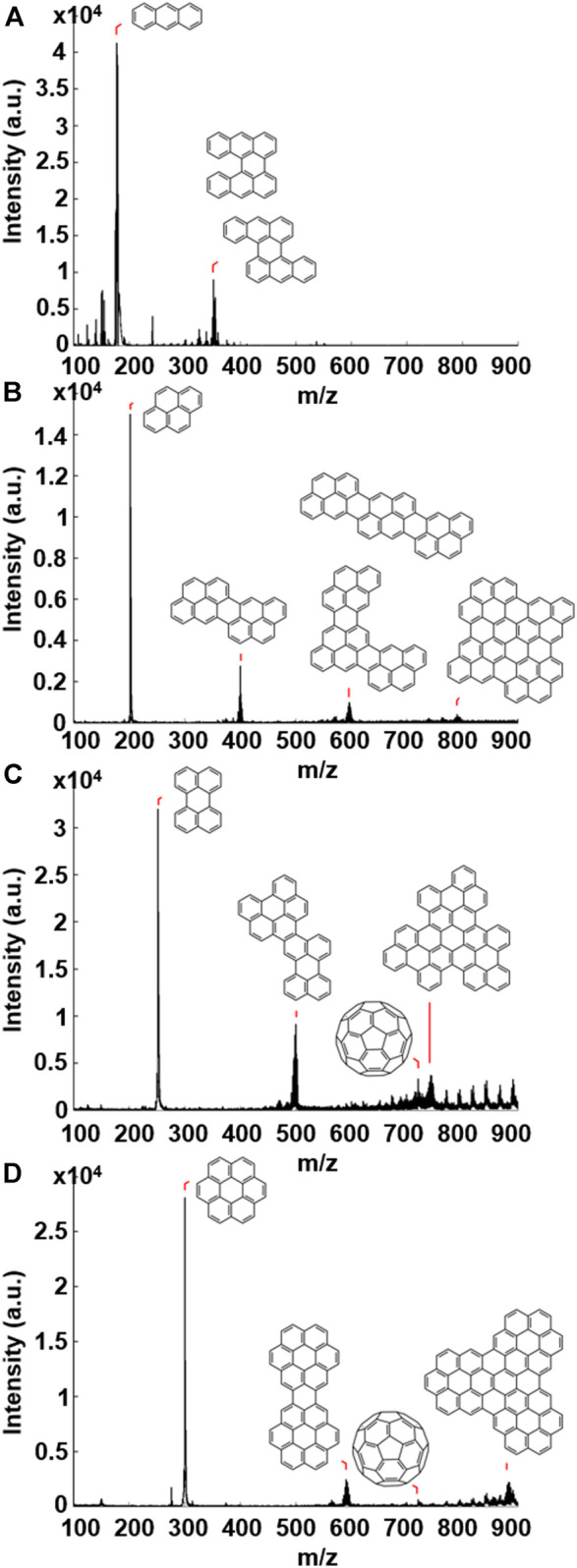
Mass spectra resulting from UV laser irradiation at high laser fluence (∼2,600 mW cm^−2^) of; **(A)** anthracene, **(B)** pyrene, **(C)** perylene and **(D)** coronene. Proposed structures of the species present are illustrated above the corresponding m/z peaks.

A thin layer of each of the four PAH samples was drop-cast onto a stainless steel sample holder, inserted into the vacuum chamber (10^–7^ mbar) and irradiated using the Nd:YAG laser (355 nm, 5 ns) of the MALDI-TOF mass spectrometer. The laser fluence was varied between ∼2,000 and ∼2,600 mW cm^−2^, which equates to a “dose rate” (comparable to that of the e-beam in TEM) of ∼34,000–∼44,000 hν nm^−2^ s^−1^ (see Supplementary Material for full details). In each case, experiments carried out at low laser fluence showed the presence of the corresponding molecular ion peaks only, indicating simply that the PAH molecules were being vaporised and ionised, as in the example of pyrene described above. At higher laser fluence the resultant mass spectra revealed the formation of larger structures; major peaks are seen at two or three times the mass of the individual PAH, minus a number of mass units. These are assigned to PAH oligomer structures formed through C-C bond formation following the loss of hydrogen atoms; this hydrogen atom loss accounts for the unit mass deficit that is observed. Similar irradiation-induced formation of PAH oligomers, specifically from coronene to form larger coronene oligomers, has been reported (Joblin et al., 1997). Figure 2 shows the resultant mass spectra following high laser fluence (∼2,600 mW cm^−2^) irradiation of **(A)** anthracene, **(B)** pyrene, **(C)** perylene and **(D)** coronene, and includes proposed structures of the oligomers formed above their corresponding mass peaks. Descriptions and m/z values of the PAH molecules and oligomers are given in Supplementary Table S2. The formation of oligomeric PAH structures under these conditions is expected to occur due to the loss of hydrogen atoms on the periphery of the PAH. This is similar to the process when the sample is irradiated under the e-beam of TEM; the loss of a hydrogen atom results in the formation of a reactive carbon radical, which can react with a neighbouring molecule to form a new C-C bond. It is important to note that a MALDI-TOF experiment with a blank graphite substrate showed no evidence of the formation of PAH oligomers or C_60_ (Supplementary Material for details).

In addition to the formation of PAH oligomers, irradiation of coronene and perylene also resulted in a distinctive signal at 720 m/z (Figure 2), corresponding to the fullerene C_60_. The formation of C_60_ from the irradiation of PAHs is important in relation to the question of C_60_ formation in astrophysical environments, since, while not altogether surprising considering the experimental conditions under which the molecule was discovered, this is a clear transformation solely from PAH precursors of defined initial structure. The results of this paper show, as demonstrated by mass spectrometric measurements and direct-space imaging, that fullerenes are able to form from PAHs under both UV and e-beam irradiation.

### Fullerene Formation

As the PAHs investigated here have fewer than sixty carbon atoms, and considering the facile formation of PAH oligomers, it is probable that C_60_ formation occurs as a result of shrinking, via the ejection of C_2_ units and hydrogen atoms, of PAH oligomers that contain at least sixty carbon atoms. An astrophysical “bottom-up” followed by “top-down” mechanism can therefore be envisaged, involving the aggregation of the initial PAH molecules to form oligomers, followed by ejection of a number of atoms and rearrangement of bonds to produce the stable fullerene structure. With this in mind, the mass spectrum peaks corresponding to ≥60 C atom oligomers of perylene and coronene were examined more closely in order to determine the precise masses and probable structures of these molecules, and to elucidate the products of intermediate mechanistic steps.

**FIGURE 3:**
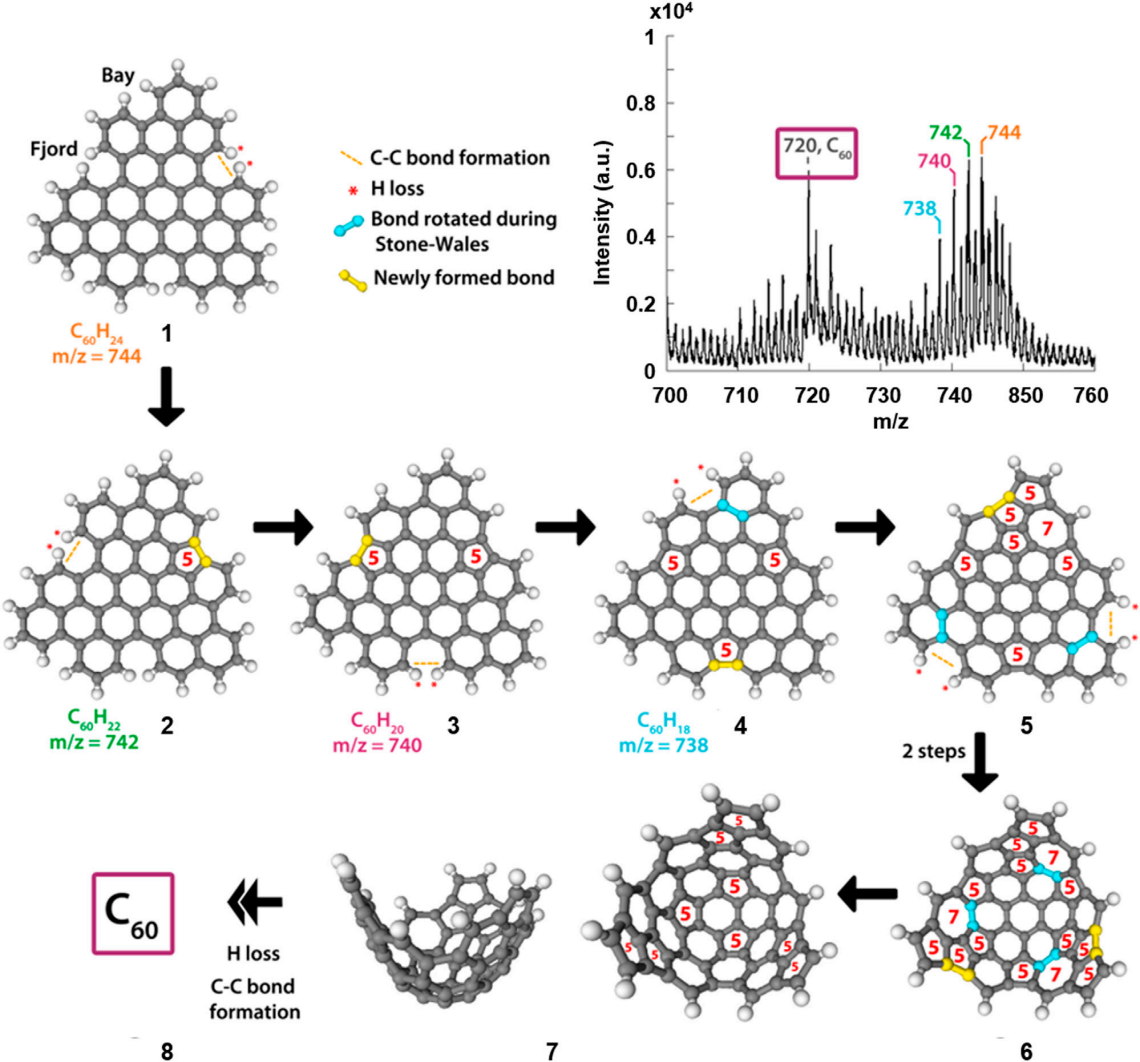
Section of the mass spectrum (Figure 2C) resulting from UV irradiation of perylene (top) and our proposed mechanistic steps based on the MALDI-TOF data and chemical intuition, showing the transformation from the perylene trimer to C_60_. The ring sizes of key parts in the structures are shown in red, and the rotated bonds and newly formed bonds are highlighted in blue and yellow respectively.

Figure 3 shows the mass spectrum resulting from the MALDI-TOF MS irradiation of perylene, in addition to proposed mechanistic steps involved in the formation of C_60_. A corresponding figure, and discussion, involving coronene is given in the (Supplementary Figure S14). The mass spectrum of Figure 3 shows a distribution of peaks that are separated by two mass units, of which the largest peak falls at m/z = 744. This peak is assigned to the perylene trimer, labelled **1** in Figure 3. N.B. Less compact, “linear” trimer species were also considered as candidates but no conceivable structure matched the molecular masses observed in the MS. Proposed structures responsible for additional peaks in the distribution (m/z = 742, 740, and 738) are labelled **2**, **3** and **4** respectively. These structures result from the loss of two hydrogen atoms, at so-called “fjord” positions in the oligomers, followed by the formation of a new C-C bond, driven by the instability of the carbon radicals that result from the initial hydrogen loss. This process provides a route for the formation of a pentagon at that position, which is a significant step as it introduces curvature into the previously planar molecule; this has been discussed by Mackie et al. (Mackie et al., 2015) for dehydrogenated PAHs in an astrophysical context.

The final mechanistic steps in Figure 3 show examples of the H atom loss, C-C bond formation, and rearrangement processes that lead to the fullerene molecule. Molecule **4** has no remaining “fjord” positions in the structure; however, if H atom loss occurred at the “bay” positions indicated in the figure, subsequent C-C bond formation would result in the creation of a strained four-membered ring. To stabilise this, a C-C bond has been highlighted that could undergo a Stone-Wales rearrangement (Stone and Wales, 1986) resulting in the formation of the structure labelled **5**. From there, there are two additional positions at which this H-loss, C-C bond formation and Stone-Wales rearrangement sequence can occur. The result of completing these steps at both positions is structure **6**. Between **6** and **7**, three potential Stone-Wales rearrangements are highlighted, the results of which would yield a structure that contains only five- and six-membered rings and with a curved shape which can then “zip-up” to form a fullerene molecule (**8**).

The mechanistic steps proposed here may not be exact, nor can the precise order of steps be determined, especially within the suggested H-loss, C-C bond formation and Stone-Wales rearrangement sequence that occurs a number of times. However, support for steps of this nature is given through the evidence seen in the detection of species by mass spectrometry. It is certainly the case that C_60_ is an extremely stable molecule, due to its lack of edges, and as such it is feasible that the loss of one or more hydrogen atoms from the edge of a PAH oligomer, and the subsequent bond formations and rearrangements, would initiate a thermodynamically driven process of which the end result is the most stable molecule, under the irradiation conditions, i.e., a fullerene.

The fact that C_60_ forms only in the case of coronene and perylene is noteworthy, and suggests a dependence on the structure of the initial PAH, or of the oligomers that are formed. An analogous result was obtained for triphenylene by Han et al. (2016) though their mechanistic interpretation differs and is focused on the “zipper” mechanism involving the rapid, concerted formation of 12 pentagon rings in one step between two overlying PAHs as advanced by Homann and co-authors. The “zipper” mechanism is based principally on topological considerations and mass spectrometric observations of hydrocarbon rich, sooting flames (Baum et al., 1992; Ahrens et al., 1994). However, such a mechanism does not explain our MS observations which reveal intermediate, discrete PAH oligomers which sequentially lose pairs of H atoms in individual steps. This could potentially be due to the nature of the energy source in our experiments, with the use of electrons or UV photons resulting in a much more stepwise mechanism compared to a thermally driven processes.

In both mechanisms the ability to form pentagons appears to be a key aspect in the propensity of a PAH to form C_60._ However, pentagon formation could also in principle occur for oligomers of anthracene and pyrene, and not only for coronene and perylene. In the case of anthracene, the highest mass oligomer that is detected following irradiation corresponds to C_28_H_16_, and if no oligomers with more than sixty carbon atoms are formed, then they cannot “shrink” to form the fullerene molecule. Pyrene, however, forms an oligomer that is detected in MS and is assigned to C_64_H_22_ (m/z = 790), so it appears that another structural aspect is important. The lack of C_60_ formation could be due to the “compactness” of the PAH oligomer following the “bottom-up” stage of the process, or it could be related to the symmetry of the oligomer; both perylene and coronene trimers have a *C*_3_ axis of rotation perpendicular to the plane of the molecule, whereas the pyrene tetramer has a *C*_2_ axis of rotation in this position. Interestingly, experiments in which an equimolar mixture of pyrene and coronene are irradiated reveal the formation of both discrete PAH oligomers consisting of fragments of both species and C_60_ implying the structure of the PAHs is important, see Supplementary Material for full details.

### Combined Transmission Electron Microscopy and MALDI-TOF MS Experiments

**FIGURE 4:**
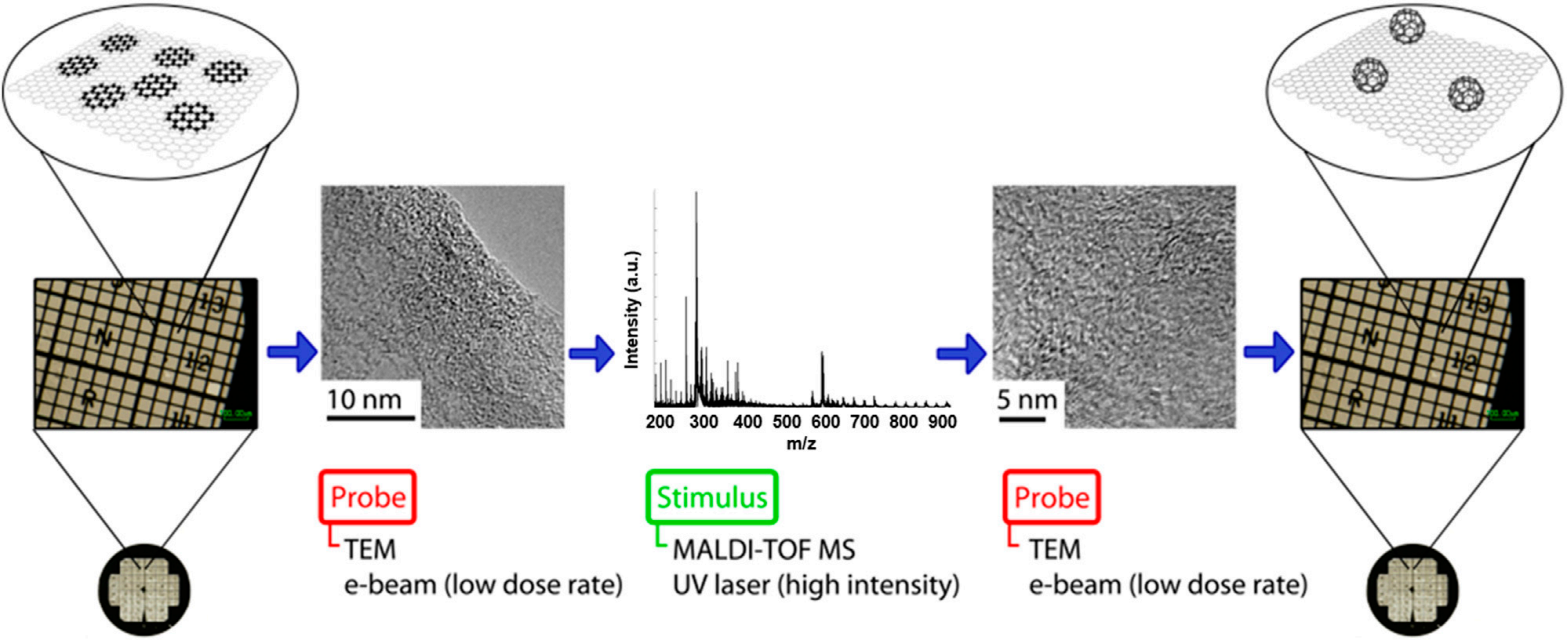
Schematic illustration of the “probe-stimulus-probe” format in which an area of coronene deposited on a TEM grid is characterized using low-dose TEM, and then irradiated using the UV laser of MALDI-TOF MS to initiate transformations within the sample. Finally, the irradiated area is characterized again, using the locators on a TEM finder grid, to view the results of the induced transformations and determine the nature of any products formed.

An experimental methodology was designed to initiate and characterize PAH transformations sequentially in order to confirm the resultant products using both microscopy and mass spectrometry. Figure 4 shows a schematic illustration detailing the steps involved in this “probe-stimulus-probe” arrangement.

**FIGURE 5:**
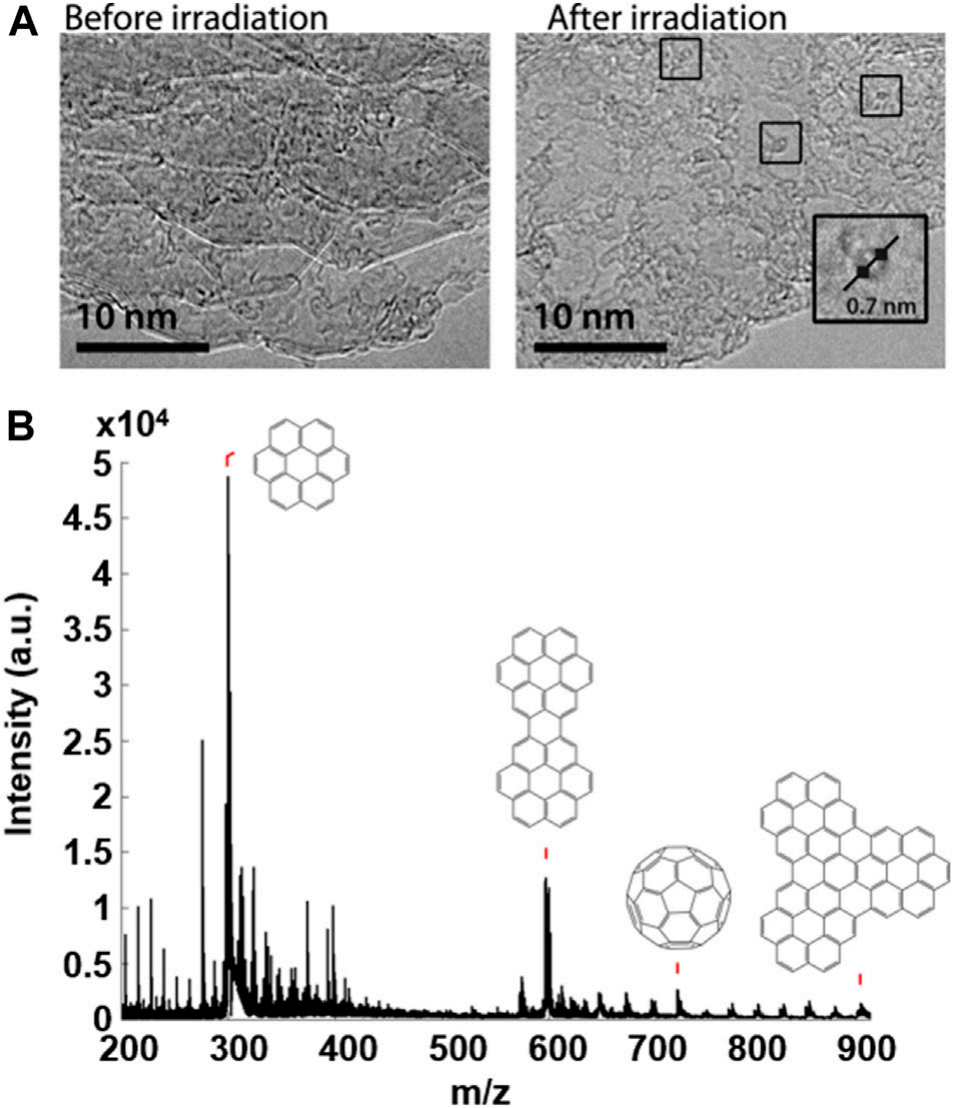
TEM micrographs **(A)** of a sample of coronene before and after laser irradiation using MALDI-TOF MS. Boxes highlight fullerene structures seen following laser irradiation along with a magnified example illustrating the diameter of the structure (0.7 ± 0.1 nm). The mass spectrum resulting from the UV irradiation of the sample **(B)** shows the oligomer structures assigned to major peaks, along with the detection of C_60_.

A sample of coronene was supported on a graphite flake, deposited on a TEM finder grid and imaged by TEM using a low dose (∼10^6^ e^−^ nm^−2^ s^−1^) of electrons and minimal exposure time to avoid transformations induced by the e-beam. The PAH sample on the grid was then irradiated with the UV laser in MALDI-TOF MS at high laser fluence (∼2,600 mW cm^−2^); the resultant mass spectrum showed coronene oligomers and C_60_. Finally, the irradiated area of the sample was reanalysed using low dose TEM to assess the effect of the UV laser irradiation. TEM micrographs obtained within the UV irradiated area of the sample reveal the formation of a number of fullerene structures not observed prior to UV irradiation. Figure 5 shows the results of this experiment, and includes the TEM micrographs obtained before and after the high laser fluence irradiation of the coronene sample, along with the mass spectrum acquired as a result of the MALDI-TOF MS (middle) part of the experiment. In addition, the sample showed far greater stability under the e-beam following UV irradiation, with structures appearing relatively static and less susceptible to e-beam-induced transformations. This indicates that the fullerene structures seen in the “after” image are in fact fullerene cages, and not remaining PAH molecules of the same diameter; if this were the case then the same sequence of peripheral hydrogen loss leading to formation of higher mass oligomers would be seen.

This experimental format can in principle be reversed, so that low laser fluence MALDI-TOF MS is used as the initial “probe,” to characterize the sample before and after irradiation, and high dose TEM is used to initiate transformations within the sample. Experiments of this type are not presented here, however, due to difficulties regarding the difference in the size of the irradiated area in TEM and MALDI-TOF MS.

## Conclusion

The existence of PAHs and fullerenes in the ISM is well established, but transformation mechanisms linking these molecules are not well understood. In this study we have demonstrated that under harsh laboratory conditions (UV laser or e-beam irradiation), medium-sized PAHs do not disintegrate, but assemble into larger carbon structures via the loss of hydrogen atoms and the condensation of reactive radical species into larger aromatic molecules with ≥60 C atoms. These undergo transformations, forming closed carbon cages — fullerenes — which are thermodynamically the most stable form of carbon at the nanoscale.

Detailed study of the molecular species in MS has shed light on mechanisms of PAH conversion to C_60_. Mechanistic steps are proposed in which the initial irradiation-induced loss of hydrogen atoms leads, via carbon radical-containing species which react with neighbouring molecules, to the formation of large planar oligomers. A second “top-down” step involves H atom loss, C-C bond formation, and rearrangements such as Stone-Wales within the planar oligomers which promote the formation of pentagonal rings, and the subsequent rolling up of the structure to form fullerene cages. Notably, for the cases considered here, high-energy ejection of a carbon atom is not required to form a pentagonal ring as is the case for zig-zag-edged graphene (Chuvilin et al., 2010).

An experimental strategy developed in this study enabled the use of MALDI-TOF MS and TEM as triggers of PAH transformations at high dose rates of UV laser or e-beam, or as analytical tools—monitoring molecular weights in MS or molecular size in TEM imagery, respectively. The cross correlation of microscopy and mass spectrometry measurements, performed here for the first time, reveals that PAHs grown from coronene and perylene undergo major transformations leading to the formation of C_60_ fullerenes while pyrene and anthracene under the same conditions form large oligomers but no fullerenes.

Our laboratory observations indicate that high-energy radiation (UV or fast electrons) triggers chemical transformations in PAHs similar to those proposed under interstellar conditions, and therefore this work provides significant support for the hypothesis that PAH molecules can be precursors to fullerene formation in the ISM. Detailed, step-by-step mechanisms have been proposed that reveal the nature of key intermediate steps within the fullerene formation process from large PAH molecules, and these reflect examples of the mechanistic steps that may be expected in the formation of C_60_ in interstellar environments.

## Data Availability

The raw data supporting the conclusion of this article will be made available by the authors, without undue reservation.

## References

[B1] Ahrens J. Bachmann M. Baum T. Griesheimer J. Kovacs R. Weilmünster P. (1994). Fullerenes and Their Ions in Hydrocarbon Flames. Int. J. Mass Spectrom. Ion Process. 138, 133–148. 10.1016/0168-1176(94)04036-2

[B2] Armand X. Herlin N. Voicu I. Cauchetier M. (1997). Fullerene Synthesis by Laser Pyrolysis of Hydrocarbons. J. Phys. Chem. Sol. 58, 1853–1859. 10.1016/S0022-3697(97)00092-9

[B3] Baum T. Löffler S. Löffler P. Weilmünster P. Homann K.-H. (1992). Fullerene Ions and Their Relation to PAH and Soot in Low-Pressure Hydrocarbon Flames. Berichte der Bunsengesellschaft für physikalische Chem. 96, 841–857. 10.1002/bbpc.19920960702

[B4] Berné O. Montillaud J. Joblin C. (2015a). Top-down Formation of Fullerenes in the Interstellar Medium. A&A 577, A133–A141. 10.1051/0004-6361/201425338 PMC4693962 26722131

[B5] Berné O. Montillaud J. Mulas G. Joblin C. (2015b). “30 Years of Cosmic Fullerenes,” in Proceedings of the Annual Meeting of the French Society of Astronomy and Astrophysics (SF2A 2015), Toulouse, June 2–5, 2015. Editors Boissier Buat V. Cambrésy L. Martins F. Petit P., 65–70. arXiv:1510.01642.

[B6] Berné O. Tielens A. G. G. M. (2012). Formation of Buckminsterfullerene (C60) in Interstellar Space. Proc. Natl. Acad. Sci. 109, 401–406. 10.1073/pnas.1114207108 22198841 PMC3258632

[B7] Björk J. Hanke F. Palma C.-A. Samori P. Cecchini M. Persson M. (2010). Adsorption of Aromatic and Anti-aromatic Systems on Graphene through π−π Stacking. J. Phys. Chem. Lett. 1 (23), 3407–3412. 10.1021/jz101360k

[B9] Botka B. Füstös M. E. Tóháti H. M. Németh K. Klupp G. Szekrényes Z. (2014). Interactions and Chemical Transformations of Coronene inside and outside Carbon Nanotubes. Small 10, 1369–1378. 10.1002/smll.201302613 24167020

[B10] Chamberlain T. W. Biskupek J. Skowron S. T. Bayliss P. A. Bichoutskaia E. Kaiser U. (2015). Isotope Substitution Extends the Lifetime of Organic Molecules in Transmission Electron Microscopy. Small 11, 622–629. 10.1002/smll.201402081 25208335

[B11] Chuvilin A. Kaiser U. Bichoutskaia E. Besley N. A. Khlobystov A. N. (2010). Direct Transformation of Graphene to Fullerene. Nat. Chem 2, 450–453. 10.1038/nchem.644 20489712

[B12] Dappe Y. J. Andersen M. Balog R. Hornekær L. Bouju X. (2015). Adsorption and STM Imaging of Polycyclic Aromatic Hydrocarbons on Graphene. Phys. Rev. B 91, 045427. 10.1103/physrevb.91.045427

[B13] Donn B. (1968). Polycyclic Hydrocarbons, Platt Particles, and Interstellar Extinction. ApJ 152, L129–L133. 10.1086/180196

[B14] Ehbrecht M. Faerber M. Rohmund F. Smirnov V. V. Stelmakh O. Huisken F. (1993). CO2-laser-driven Production of Carbon Clusters and Fullerenes from the Gas Phase. Chem. Phys. Lett. 214, 34–38. 10.1016/0009-2614(93)85451-S

[B15] Fujihara M. Miyata Y. Kitaura R. Nishimura Y. Camacho C. Irle S. (2012). Dimerization-Initiated Preferential Formation of Coronene-Based Graphene Nanoribbons in Carbon Nanotubes. J. Phys. Chem. C 116, 15141–15145. 10.1021/jp3037268

[B16] Galué H. Á. (2014). Decoding the Infrared Signatures of Pyramidal Carbons in Graphenic Molecular Nanostructures of Interstellar Origin. Chem. Sci. 5, 2667–2676. 10.1039/c4sc00890a

[B17] Goroff N. S. (1996). Mechanism of Fullerene Formation. Acc. Chem. Res. 29, 77–83. 10.1021/ar950162d

[B18] Han J. Y. Choi T. S. Kim S. Lee J. W. Ha Y. Jeong K. S. (2016). Probing Distinct Fullerene Formation Processes from Carbon Precursors of Different Sizes and Structures. Anal. Chem. 88, 8232–8238. 10.1021/acs.analchem.6b02076 27434606

[B19] Heya A. Oonuki T. Utimi R. Kanda K. Yamasaki R. Sumitomo K. (2020). Graphene Synthesis from Pentacene by Soft X-ray Irradiation. Thin Solid Films 713, 138365–138410. 10.1016/j.tsf.2020.138365

[B20] Ishii Y. Sakashita T. Kawasaki S. Kato H. Takatori M. (2011). Fusing Treatment of Pentacenes: toward Giant Graphene-like Molecule. Mat Express 1, 36–42. 10.1166/mex.2011.1005

[B21] Joblin C. Masselon C. Boissel P. de Parseval P. Martinovic S. Muller J.-F. (1997). Simulation of Interstellar Aromatic Hydrocarbons Using Ion Cyclotron Resonance. Preliminary Results. Rapid Commun. Mass. Spectrom. 11, 1619–1623. 10.1002/(sici)1097-0231(199709)11:14<1619::aid-rcm995>3.0.co;2-p

[B22] Kroto H. W. Heath J. R. O’Brien S. C. Curl R. F. Smalley R. E. (1985). C60: Buckminsterfullerene. Nature 318, 162–163. 10.1038/318162a0

[B23] Linnartz H. Cami J. Cordiner M. Cox N. L. J. Ehrenfreund P. Foing B. (2020). C60+ as a Diffuse Interstellar Band Carrier; a Spectroscopic story in 6 Acts. J. Mol. Spectrosc. 367 (8), 111243. 10.1016/j.jms.2019.111243-0022-2852

[B24] Liu S. Lu Y.-J. Kappes M. M. Ibers J. A. (1991). The Structure of the C60 Molecule: X-Ray Crystal Structure Determination of a Twin at 110 K. Science 254 (5030), 408–410. 10.1126/science.254.5030.408 17742229

[B25] Mackie C. J. Peeters E. Bauschlicher Jr. C. W. Cami J. (2015). Characterizing the Infrared Spectra of Small, Neutral, Fully Dehydrogenated Polycyclic Aromatic Hydrocarbons. ApJ 799, 131–141. 10.1088/0004-637X/799/2/131

[B26] Maier J. P. Campbell E. K. (2016). Pathway to the Identification of C 60 + in Diffuse Interstellar Clouds. Phil. Trans. R. Soc. A. 374 (12), 20150316. 10.1098/rsta.2015.0316 27501976 PMC4978740

[B27] Markevich A. Kurasch S. Lehtinen O. Reimer O. Feng X. Müllen K. (2015). Electron Beam Controlled Covalent Attachment of Small Organic Molecules to Graphene. Nanoscale 8, 2711. 10.1039/c5nr07539d 26757842

[B28] Mirzayev R. Mustonen K. Monazam M. R. A. Mittelberger A. Pennycook T. J. Mangler C. (2017). Buckyball Sandwiches. Sci. Adv. 3 (6)), e1700176. 10.1126/sciadv.1700176 28630925 PMC5466370

[B29] Osterodt J. Zett A. Vögtle F. (1996). Fullerenes by Pyrolysis of Hydrocarbons and Synthesis of Isomeric Methanofullerenes. Tetrahedron 52, 4949–4962. 10.1016/0040-4020(96)00103-2

[B30] Roberts K. R. G. Smith K. T. Sarre P. J. (2012). Detection of C60 in Embedded Young Stellar Objects, a Herbig Ae/Be star and an Unusual post-asymptotic Giant branch star. Monthly Notices R. Astronomical Soc. 421, ‏3277–3328. 10.1111/j.1365-2966.2012.20552.x

[B31] Scott L. T. Boorum M. M. McMahon B. J. Hagen S. Mack J. Blank J. (2002). A Rational Chemical Synthesis of C60. Science 295, 1500–1503. 10.1126/science.1068427 11859187

[B32] Semeniuk M. Yi Z. Poursorkhabi V. Tjong J. Jaffer S. Lu Z.-H. (2019). Future Perspectives and Review on Organic Carbon Dots in Electronic Applications. ACS Nano 13, 6224–6255. 10.1021/acsnano.9b00688 31145587

[B33] Skowron S. T. Chamberlain T. W. Biskupek J. Kaiser U. Besley E. Khlobystov A. N. (2017). Chemical Reactions of Molecules Promoted and Simultaneously Imaged by the Electron Beam in Transmission Electron Microscopy. Acc. Chem. Res. 50 (8), 1797–1807. 10.1021/acs.accounts.7b00078 28696097

[B34] Stone A. J. Wales D. J. (1986). Theoretical Studies of Icosahedral C60 and Some Related Species. Chem. Phys. Lett. 128, 501–503. 10.1016/0009-2614(86)80661-3

[B35] Talyzin A. V. Anoshkin I. V. Krasheninnikov A. V. Nieminen R. M. Nasibulin A. G. Jiang H. (2011). Synthesis of Graphene Nanoribbons Encapsulated in Single-Walled Carbon Nanotubes. Nano Lett. 11, 4352–4356. 10.1021/nl2024678 21875092

[B36] Talyzin A. V. Luzan S. M. Leifer K. Akhtar S. Fetzer J. Cataldo F. (2011). Coronene Fusion by Heat Treatment: Road to Nanographenes. J. Phys. Chem. C 115, 13207–13214. 10.1021/jp2028627

[B37] Taylor R. Langley G. J. Kroto H. W. Walton D. R. M. (1993). Formation of C60 by Pyrolysis of Naphthalene. Nature 366, 728–731. 10.1038/366728a0

[B38] Tian P. Tang L. Teng K. S. Lau S. P. (2018). Graphene Quantum Dots from Chemistry to Applications. Mater. Today Chem. 10, 221–258. 10.1016/j.mtchem.2018.09.007

[B39] Tielens A. G. G. M. (2008). Interstellar Polycyclic Aromatic Hydrocarbon Molecules. Annu. Rev. Astron. Astrophys. 46, 289–337. 10.1146/annurev.astro.46.060407.145211

[B40] Tielens A. G. G. M. (2013). The Molecular Universe. Rev. Mod. Phys. 85, 1021–1081. 10.1103/revmodphys.85.1021

[B41] Weippert J. Hauns J. Bachmann J. Greisch J.-F. Narita A. Müllen K. (2020). Oligomerization of Dehydrogenated Polycyclic Aromatic Hydrocarbons on Highly Oriented Pyrolytic Graphite. J. Phys. Chem. C 124, 8236–8246. 10.1021/acs.jpcc.0c00883

[B42] Yan Y. Gong J. Chen J. Zeng Z. Huang W. Pu K. (2019). Recent Advances on Graphene Quantum Dots: from Chemistry and Physics to Applications. Adv. Mater. 31 (22pp), 1808283. 10.1002/adma.201808283 30828898

[B43] Zhen J. Castellanos P. Paardekooper D. M. Linnartz H. Tielens A. G. G. M. (2014). Laboratory Formation of Fullerenes from PAHs: Top-Down Interstellar Chemistry. ApJ 797, L30–L34. 10.1088/2041-8205/797/2/l30

[B44] Zhen J. Chen T. Tielens A. G. G. M. (2018). Laboratory Photochemistry of Pyrene Clusters: an Efficient Way to Form Large PAHs. ApJ 863 (6pp), 128. 10.3847/1538-4357/aad240

[B45] Zhen J. (2019). Laboratory Formation of Large Molecules in the Gas Phase. A&A 623 (5pp), A102. 10.1051/0004-6361/201834847

[B46] Zhen J. Zhang W. Yang Y. Zhu Q. Zhu Q. F. (2019). Laboratory Formation and Photo-Chemistry of Ionic HBC/anthracene Clusters in the Gas Phase. Monthly Notices R. Astronomical Soc. 486, 3259–3265. 10.1093/mnras/stz1095

