# Supplementary material for: Piecing Together Large Polycyclic Aromatic Hydrocarbons and Fullerenes: A Combined ChemTEM Imaging and MALDI-ToF Mass Spectrometry Approach
Source: Front Chem. 2021 Jun 14;9:700562. doi: 10.3389/fchem.2021.700562 (PMC8238087; doi:10.3389/fchem.2021.700562)
Supplement: Supplementary file 1 [file DataSheet1.pdf]

Piecing together large polycyclic aromatic hydrocarbons and fullerenes: A combined ChemTEM imaging and MALDI-ToF mass spectrometry approach

R. K. E. Gover, T. W. Chamberlain, P. J. Sarre and A. N. Khlobystov

Supporting information

S1. Experimental

PAH samples (coronene, perylene, pyrene and anthracene) were obtained from Sigma-Aldrich and used as received. For the TEM samples, graphite flakes (~ 1 mg, Fisher Scientific: Graphite powder, Supplier: Fisher Scientific, Code: G/0900/60, Batch: 0438775, Cas No.: 7782-42-5) were dispersed in HPLC-grade isopropyl alcohol (IPA) (4 mL) using an ultrasonic bath, drop-cast (25 drops) onto lacey carbon coated copper TEM grids (AGAR) and allowed to dry. The PAH sample (~ 0.5 mg) was then dispersed in HPLC-grade IPA (4 mL) using an ultrasonic bath and drop-cast (25 drops) on top of the graphite flakes. The grid was then dried under a flow of N_2 for 1 min. For the MALDI-TOF mass spectrometry (MS) experiments, the PAH sample (~ 1 mg) was dispersed in acetonitrile (2 mL) using an ultrasonic bath and was then drop-cast (1 drop) onto a stainless steel Multiprobe Adapter MALDI target. For the combined methodology experiments, a holey carbon TEM finder grid (Agar Scientific) was used for sample preparation. The grid was then secured to a stainless steel Multiprobe Adapter MALDI target using adhesive tape for laser irradiation. Finally, the grid was removed from the MALDI target plate and TEM imaging performed on the resultant sample.

TEM imaging of the samples was carried out using a JEOL-2100F TEM instrument at an accelerating voltage of 100 kV. When using TEM as a probe the e-beam was spread in order to keep the total dose of electrons as low as possible to avoid inducing changes in the sample.

UV laser irradiation experiments were carried out using a Bruker Ultraflex III MALDI-TOF mass spectrometer in positive reflection mode. This was equipped with a pulsed Nd:YAG 355 nm laser with 5 ns-duration pulses of ~ 500 μJ pulse^{-1}. Each spectrum was acquired from an average of 200 shots. Samples were irradiated in steps of laser power of 282 mW cm^{-2}, in the range 2000 – 2600 mW cm^{-2}.

S2. Electron-beam irradiation of PAHs using TEM

Included in this section are time series images taken during the irradiation of each of the PAH samples with the e-beam in TEM (Figures S1 – S4). In all cases it is seen that over time the species increase in size as PAH molecules react to form larger PAH oligomers. In some cases, fullerene molecules can be seen to have formed. Videos of the time series for each PAH are also available.

A number of molecules were selected from the first and final frames of each of the time series, and were measured in terms of the largest distance from one edge of the molecule to the other, before calculating the mean of the values. These measurements are presented in Table S1, which shows the average sizes of a representative sample of oligomers at the beginning and end of each time series, and demonstrates an increase in size in each case, supporting the idea of growth and formation of oligomeric species. This increase is not always as large as might be expected. For example, if a PAH 'oligomer' has formed from two individual molecules, then an increase in size of 100 % would be expected and this is clearly not seen. There are two possible explanations for this result. Firstly, the initial frame of the time series is not likely to be exactly representative of a sample of individual PAH molecules, as the sample is exposed to the e-beam for a short period of time during the focussing of the beam and before the first image is recorded. During this time the PAH molecules are likely to have started to undergo transformations, and measuring species that are already larger than individual PAH molecules would account for a smaller overall increase in size over time. Additionally, the smaller-than-expected size increase could be explained if the oligomer structures, once formed, 'curled' at

their edges. This would happen if, for example, one or more five-membered rings formed at the edge of the structure causing a curvature at those positions and lifting the edges out-of-plane; the projected image of one of these molecules would then appear smaller than the molecule actually is, suggesting, therefore, that the formed species are only marginally larger than the original PAH molecules. A similar effect is seen in the experimental and theoretical investigations into the formation of fullerenes from graphene flakes (Chuvilin et al., 2010; Santana et al., 2013), in which intermediate species with curved edges were seen as shapes that are smaller than the original graphene flake, before finally forming a fullerene molecule.

Table S1. Average sizes of graphitic islands in the first and final frames taken during e-beam exposure, demonstrating the evolution of the species in terms of an increase in size over time.

	Average size (nm)		
Molecule	First	Final	Percentage size increase
Anthracene	1.06	1.44	36 %
Pyrene	1.76	1.81	3 %
Perylene	1.01	1.11	10 %
Coronene	1.21	1.92	59 %

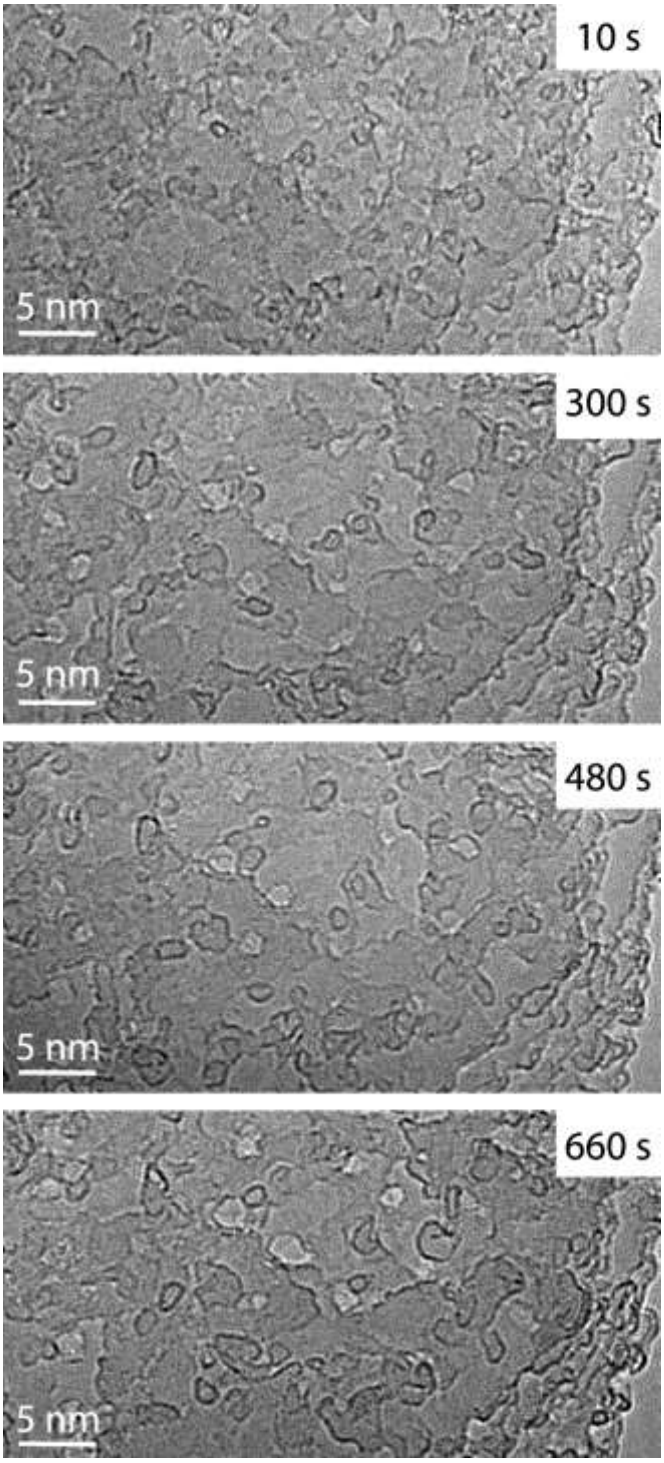

Figure S1. TEM micrographs showing the evolution of PAH oligomers during the irradiation of anthracene with a 100 keV e-beam.

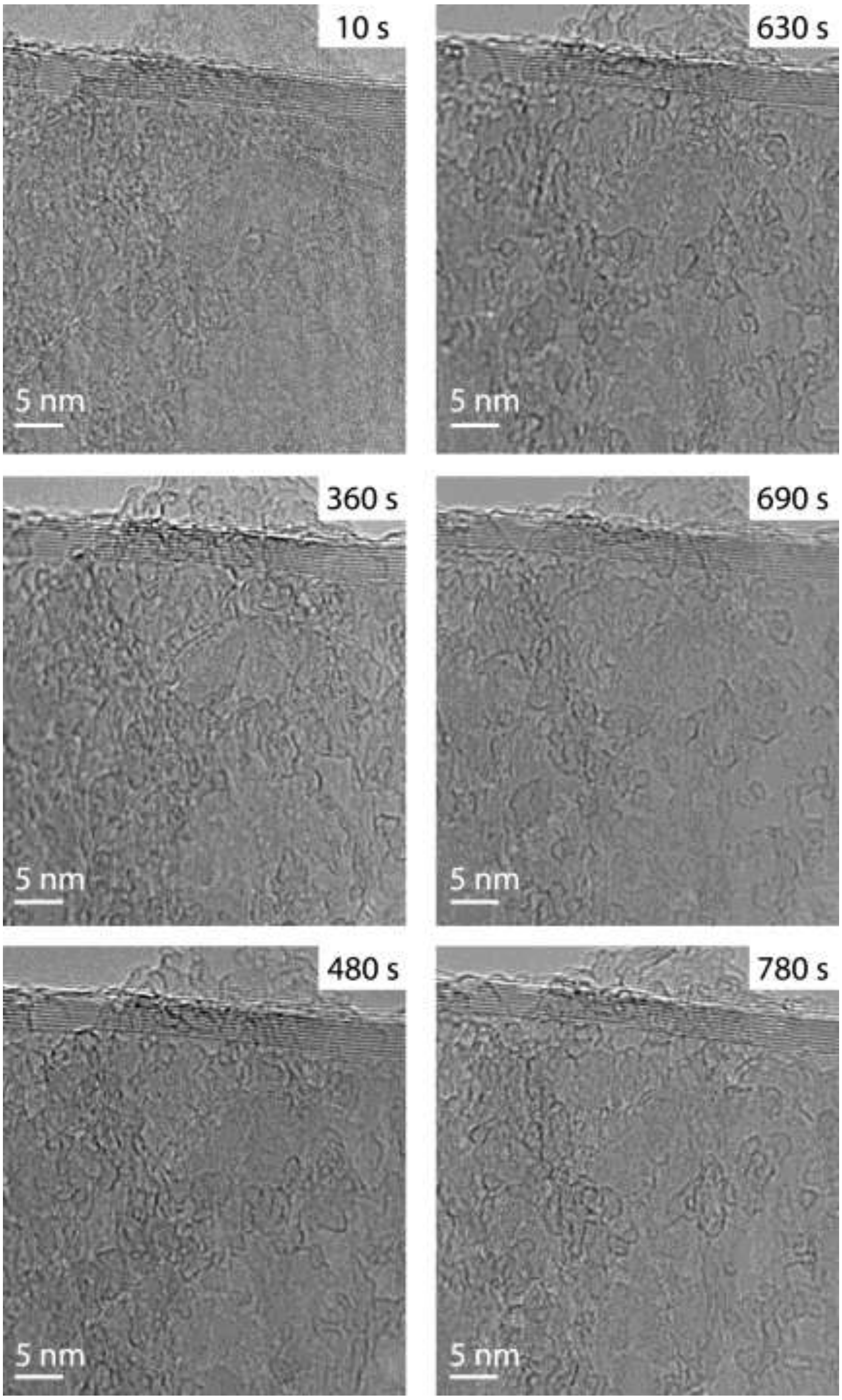

Figure S2. TEM micrographs showing the evolution of PAH oligomers during the irradiation of pyrene with a 100 keV e-beam.

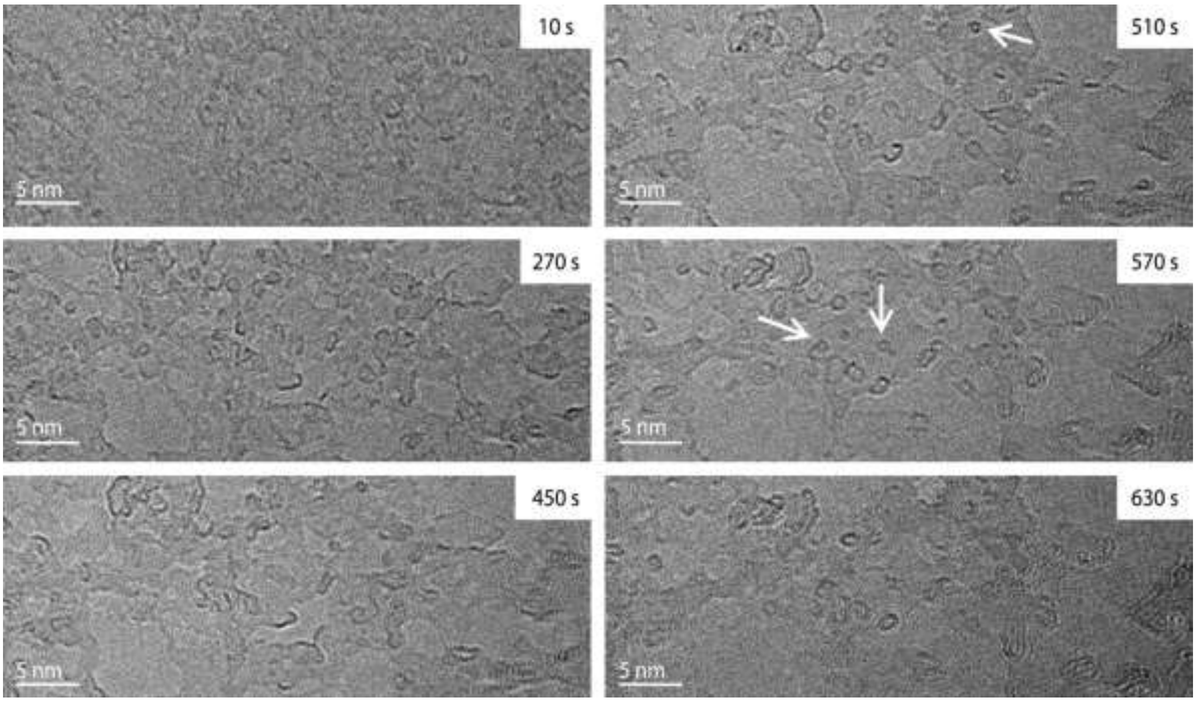

Figure S3. TEM micrographs showing the evolution of PAH oligomers during the irradiation of perylene with a 100 keV e-beam. Examples of circular, fullerene structures formed as a result of prolonged irradiation of perylene are highlighted with white arrows.

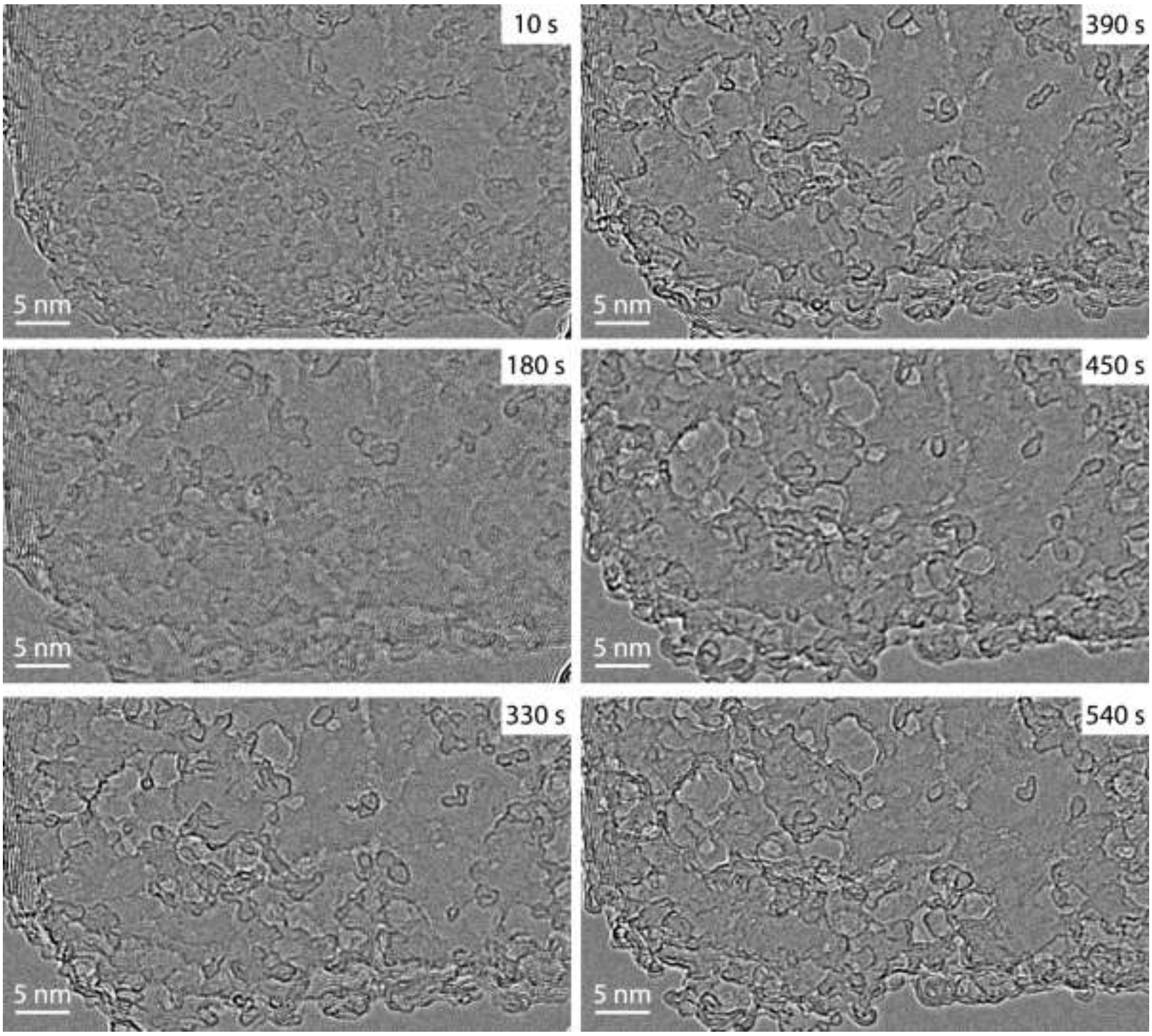

Figure S4. TEM micrographs showing the evolution of PAH oligomers during the irradiation of coronene with a 100 keV e-beam.

S3. UV irradiation of PAHs using MALDI-TOF MS

Figure S5 contains mass spectra resulting from the irradiation of a sample of pyrene at different values of laser fluence. With increasing laser fluence, higher mass species are detected indicating their formation as a result of UV irradiation. Similar observations were made for all four PAHs. Table S2 includes descriptions and m/z values of the four PAH molecules and the oligomers that are formed from each of these as a result of UV irradiation, as detected using mass spectrometry.

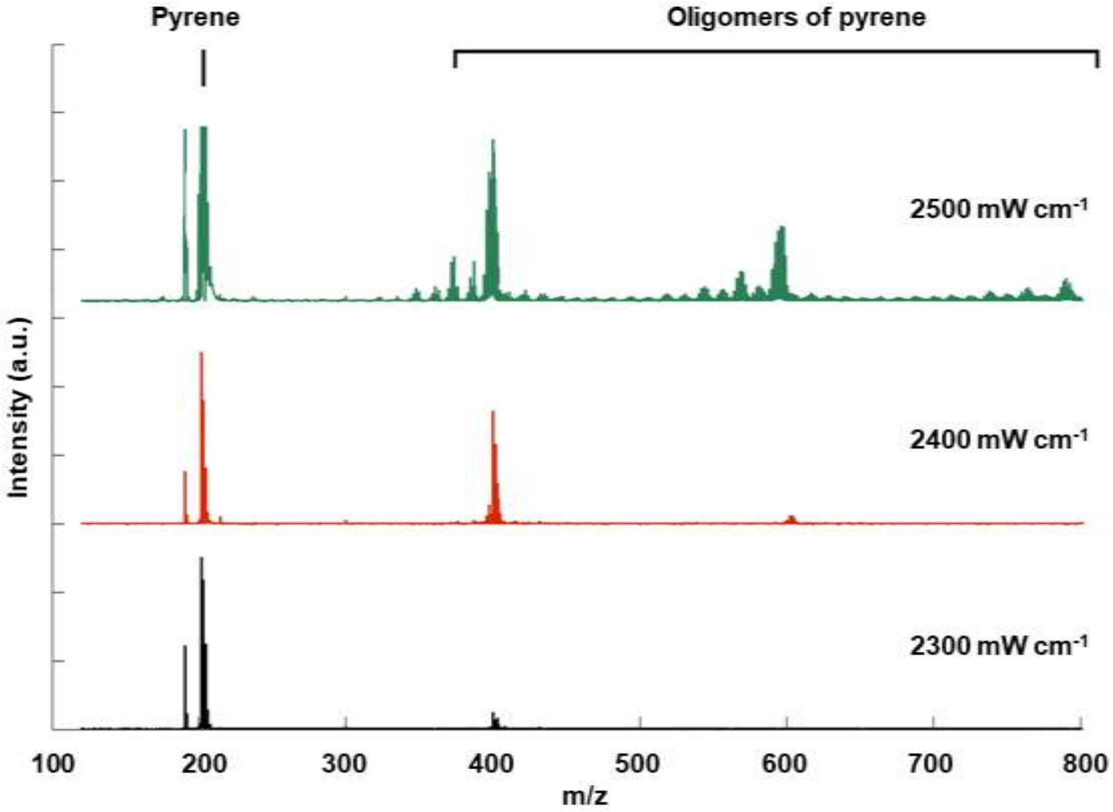

Figure S5. Mass spectra resulting from irradiation of pyrene (m/z = 202) using the UV laser of MALDI-TOF MS at the following values of laser fluence: 2300 mW cm^{-2} (bottom), 2400 mW cm^{-2} (middle) and 2500 mW cm^{-2} (top). On increasing the laser fluence, higher mass species are detected indicating their formation via laser-induced transformations within the initial PAH sample.

Table S2. m/z values and assignments of the major peaks seen in Figure 2 of the main article, along with descriptions of the predicted structures, which are shown pictorially in Figure 2 of the main article.

PAH	m/z of peak	Assignment	Description
Anthracene	178	$C_{14}H_{10}$	Molecular ion.
	352	$C_{28}H_{16}$	'Dimer' structure containing two anthracene units each having lost two hydrogen atoms.
Pyrene	202	$C_{16}H_{10}$	Molecular ion.
	400	$C_{32}H_{16}$	'Dimer' structure containing two pyrene units each having lost two hydrogen atoms.
	598	$C_{48}H_{22}$	'Trimer' structure containing three pyrene units with overall loss of eight hydrogen atoms.
	790	$C_{64}H_{22}$	Condensed 'tetramer' structure containing four pyrene units with overall loss of eighteen hydrogen atoms.
Perylene	252	$C_{20}H_{12}$	Molecular ion.
	500	$C_{40}H_{20}$	'Dimer' structure containing two perylene units each having lost two hydrogen atoms.
	720	C_{60}	C_{60} fullerene.
	744	$C_{60}H_{24}$	Condensed 'trimer' structure containing three perylene units with overall loss of twelve hydrogen atoms.
Coronene	300	$C_{24}H_{12}$	Molecular ion.
	596	$C_{48}H_{20}$	'Dimer' structure containing two coronene units each having lost two hydrogen atoms.
	720	C_{60}	C_{60} fullerene.
	888	$C_{72}H_{24}$	Condensed 'trimer' structure containing three coronene units with overall loss of twelve hydrogen atoms.

Effect of laser power

An experiment was carried out in which the number of laser pulses was reduced in the irradiation of coronene and of perylene, while the laser fluence was kept the same as for the main UV irradiation experiments (~ 2600 mW cm^{-2}). The resultant mass spectra were compared with those from experiments in which two hundred pulses were used, and the comparisons are given in Figure S6. In both cases, the mass spectra resulting from one laser pulse were less intense than those resulting from two hundred laser pulses, by about two orders of magnitude. In order to compare these results, all mass spectra presented in Figure S6 have been scaled to the same intensity of the main molecular ion peak of the 200-pulse mass spectra for each PAH.

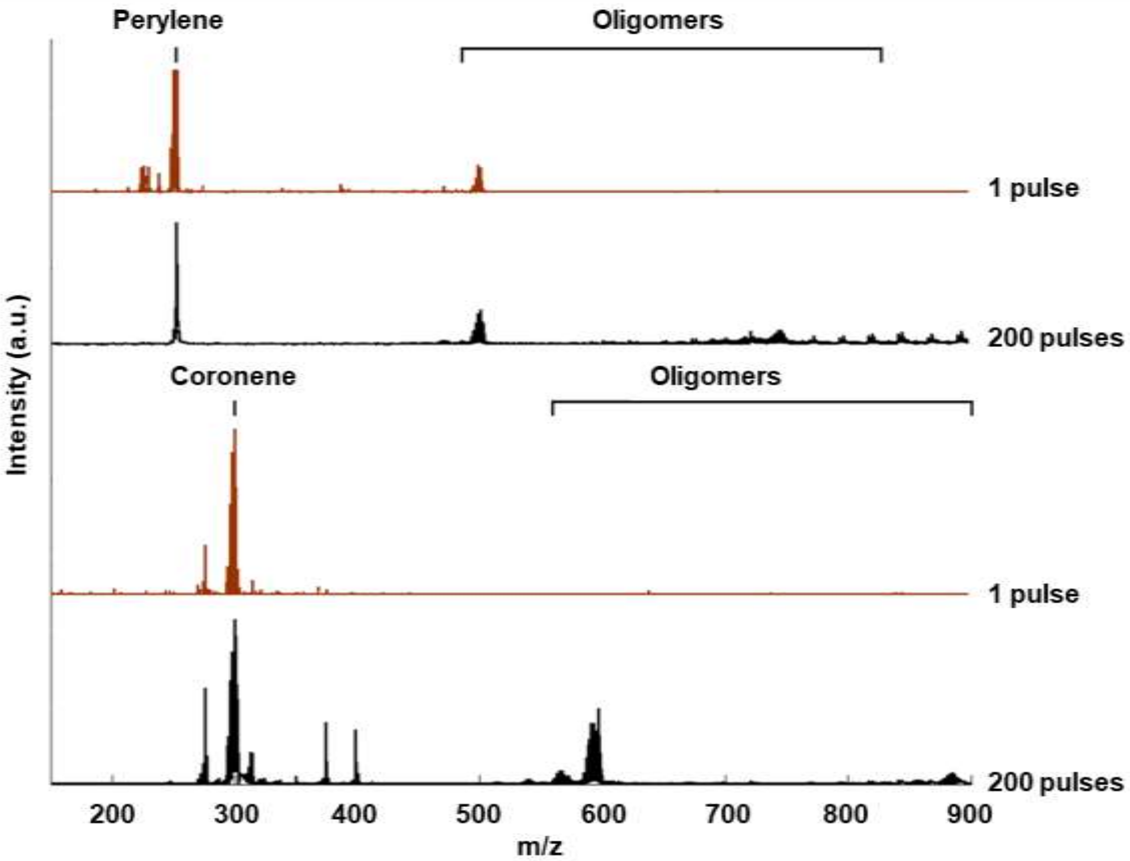

Figure S6. Mass spectra resulting from MALDI-TOF MS experiments on perylene (top) and coronene (bottom), comparing the results of one laser pulse with two hundred successive pulses, revealing the absence of formation of PAH oligomers with only one laser pulse. The molecular ion peak for each of the '1 pulse' spectra has been normalised to that of the '200 pulse' spectra by multiplying by a factor of approximately 100.

S4. Mixtures of PAHs

In astrophysical environments, the population of PAHs would include many different PAH molecules – not just a single type of structure. For this reason, TEM and MALDI-TOF MS irradiation experiments were carried out on mixtures of PAHs in order to determine whether there are any significant changes in the behaviour of the molecules, or in the products formed.

TEM of a mixture of PAH molecules

A 1:1 equimolar mixture of pyrene and coronene was dispersed and deposited onto a TEM grid, following the same procedure that was used for individual PAH experiments. As for the experiments using single species, the mixtures of PAHs were supported on graphite flakes, the experiment was run for approximately 30 mins, and images were taken periodically to view the changes to the sample over time. Once again, the TEM experiment was carried out at an accelerating voltage of 100 kV, at which the ejection of the hydrogen atoms from the PAH molecules will occur. As such, it was expected that a similar behaviour would be observed as in the experiments carried out on each individual PAH sample, i.e. the growth of PAH aggregates and oligomers. The time-series of images acquired from this experiment is given in Figure S7, in which it can be seen that a very similar behaviour to that of a sample of a single type of PAH is indeed observed. The first image of the time series in Figure S7 shows

the sample containing no well-defined structures which, as time goes on, becomes clearer and contains many defined, discrete species. This suggests that the aggregation of PAHs and the formation of larger oligomers is not exclusive to a sample containing one type of PAH.

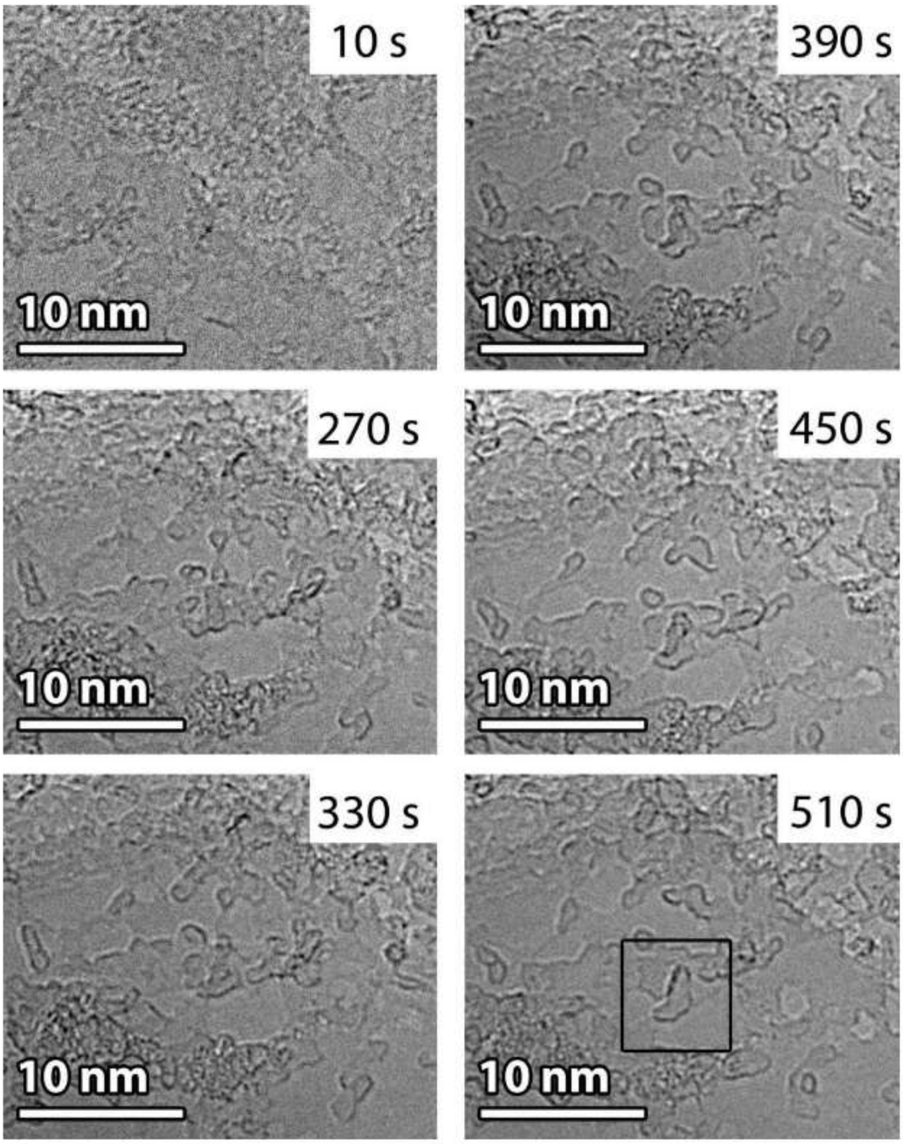

Figure S7. TEM micrographs showing the evolution of PAH oligomers during the irradiation of a mixture of pyrene and coronene with a 100 keV e- beam. The black box in the final image of the time series indicates the shape that is shown in Figure S8.

In the final image of Figure S7 (at 510 s), an example of a PAH oligomer is highlighted in the black box. This shape is presented at a higher magnification in Figure S8 and is labelled with some of its measurements. From the measurements taken of this particular shape in the TEM image, a structure containing coronene and pyrene units was constructed which closely matched its size, and which is shown also in Figure S8. While the structure given in Figure S8 may not be the exact molecule that is seen in the TEM micrograph, it provides an example to guide the eye and to understand how the PAH molecules can interact with each other following loss of their hydrogen atoms.

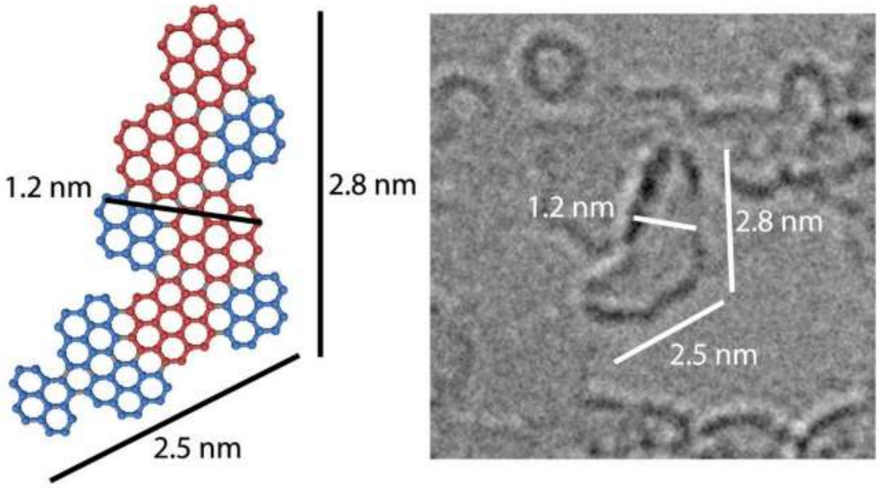

Figure S8. Example of a PAH oligomer or aggregate seen in the TEM irradiation of a mixture of coronene and pyrene. The TEM image used (right) is that which is outlined in the black box in Figure S7. The measurements of the oligomer are shown, and a corresponding structure is given with the same measurements (left), which is made up of coronene units (red) and pyrene units (blue).

MALDI-TOF MS of a mixture of PAH molecules

A thin layer of a mixture of coronene and perylene was deposited onto the MALDI-TOF MS target plate, and a UV irradiation experiment was carried out as detailed for single PAH experiments. The mass spectrum resulting from the UV irradiation of this mixture, at a laser fluence of ~ 2600 mW cm^{-2}, is shown in Figure S9. Included in the figure are structures assigned to the major peaks. The mass spectrum shown in Figure S9 seems, for the most part, to be a superposition of the mass spectra of the individual PAHs; there are seen the molecular ion peaks of both perylene (m/z = 252) and coronene (m/z = 300), along with each of their ‘dimer’ molecules (perylene dimer m/z = 500, coronene dimer m/z = 596). One additional peak is seen in the mass spectrum that falls at m/z = 548, which can be assigned to a molecule with one coronene and one perylene unit, each having lost two hydrogen atoms. Two possible structures for this mixed-dimer molecule are shown in the grey box in Figure S9, one of which contains two fjord regions. It cannot be determined which of these mixed-dimer structures is the one being detected – or if it is in fact both – but there are additional peaks that fall at m/z = 546 and 544, which may well arise from the loss of two and four fjord hydrogen atoms, respectively. In addition, it can be seen in Figure S9 that C_{60} is detected, indicating that its formation under these conditions is not exclusive to samples containing only one type of PAH as a precursor; this is of significance in an astrophysical context as the ISM clearly contains a wide variety of PAHs.

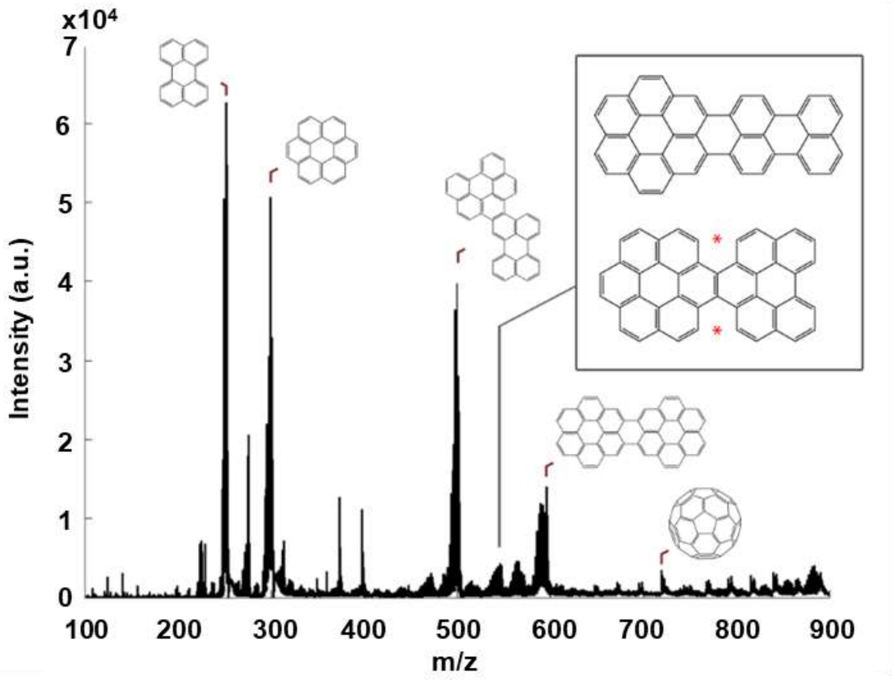

Figure S9. Mass spectrum resulting from UV irradiation of a mixture of coronene and perylene, at a laser fluence of ~ 2600 mW cm^{-2}. Proposed structures of the species formed are given above the corresponding mass peaks, including new possible hybrid structures in the grey box. Red asterisks indicate fjord positions.

It was observed that both coronene and perylene individually exhibit fullerene formation under UV irradiation, and, as shown in Figure S9, the same is seen when a mixture of the two is used. The same MALDI-TOF MS irradiation experiment was carried out on a mixture of coronene and pyrene, the latter being a PAH molecule that does not form C_{60} on its own, and the resultant mass spectrum is given in Figure S10. It can be seen that similar results to the coronene/perylene mixture are obtained. Peaks are seen that correspond to pyrene (m/z = 202), coronene (m/z = 300), and the 'dimers' of each (pyrene dimer m/z = 400, coronene dimer m/z = 596). Additionally, there is a peak at m/z = 498 that corresponds to a mixed-dimer structure, and C60 is detected at m/z = 720.

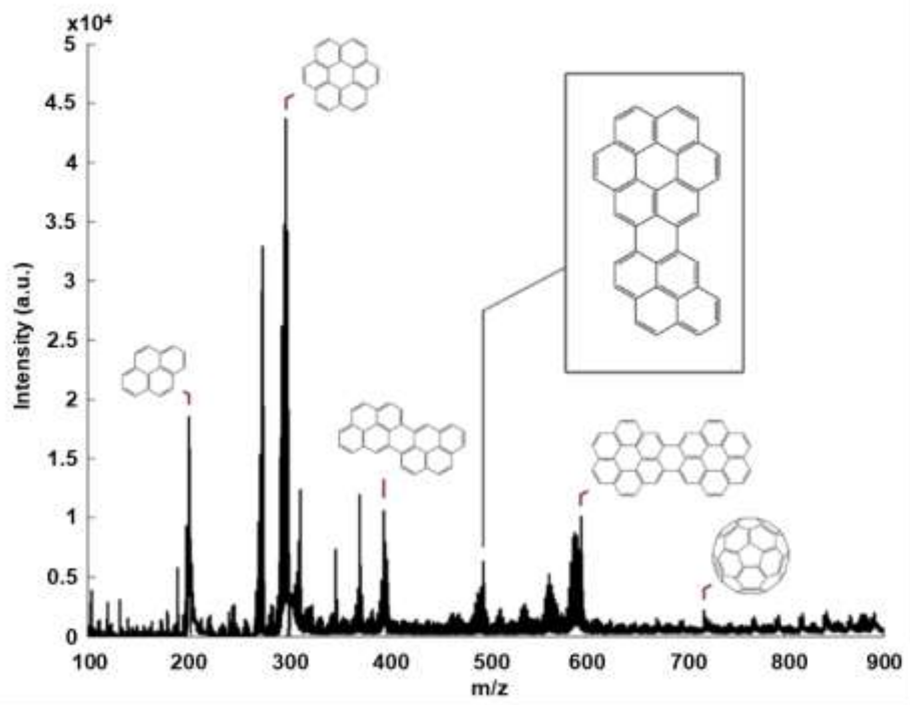

Figure S10. Mass spectrum resulting from UV irradiation of a mixture of coronene and pyrene, at a laser fluence of ~ 2600 mW cm^{-2}. Proposed structures of the species formed are given above the corresponding mass peaks, including a new possible hybrid structure in the grey box.

The results of the TEM and MALDI-TOF MS experiments carried out on mixtures of PAHs confirm that in a population of PAHs containing many structures, the behaviour of these molecules is likely to be very similar to that seen for individual samples of different molecules. Upon dehydrogenation, or loss of individual hydrogen atoms, these molecules will react with others close by to form new C-C bonds and in doing so will build up to form larger PAH structures. The results of UV irradiation of mixtures of PAHs using MALDITOF MS particularly demonstrate that while, individually, certain PAH molecules do not undergo transformations that result in the formation of C_{60}, the inclusion of these PAHs in mixtures does not negatively impact on fullerene formation. This is a positive result in the context of astrophysical fullerene formation, as it is a slightly closer representation of astrophysical PAH populations, which would contain more than one type of PAH.

S5. Graphitic flake controls

It is important to note that e-beam irradiation of control samples containing only blank graphite flake substrates showed no evidence of the formation of oligomers or C_{60} (Figure S11), providing additional support for PAHs as the sole precursors to fullerene formation in these experiments.

An initial control experiment was carried out in which graphite flakes were deposited onto a MALDI target plate and irradiated using the UV laser at the same laser fluence (~ 2600 mW cm^{-2}) as was used in the main experiment. This was to eliminate the possibility that C_{60}, or indeed any peaks falling where PAH oligomers are expected, might be formed from graphite flakes in these conditions. The resultant mass spectrum is given in Figure S12, and it is seen that there is no C_{60} detected. Additionally, no peaks are seen in the spectrum that fall at m/z values of the PAH oligomers. As such, PAH oligomers and

fullerenes detected in the combined TEM and MALDI-TOF MS experiment can be attributed with confidence to structures formed from the PAH molecules themselves.

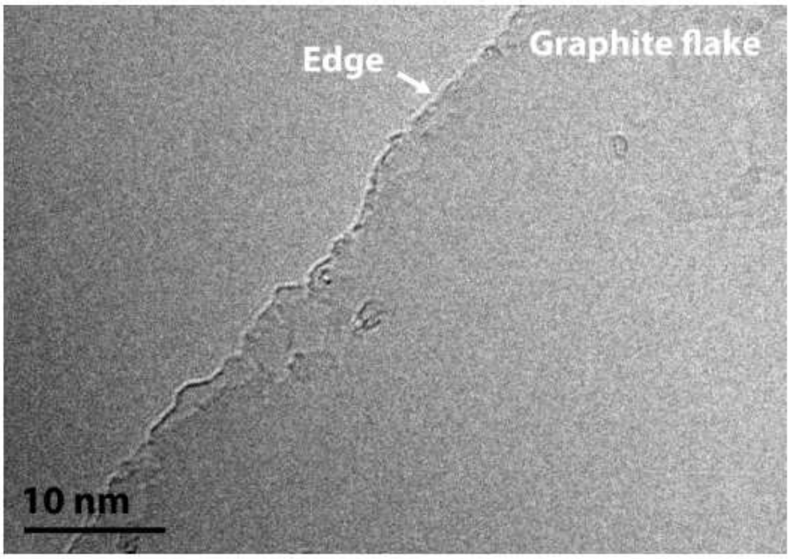

Figure S11. TEM micrograph of blank graphite flake substrate.

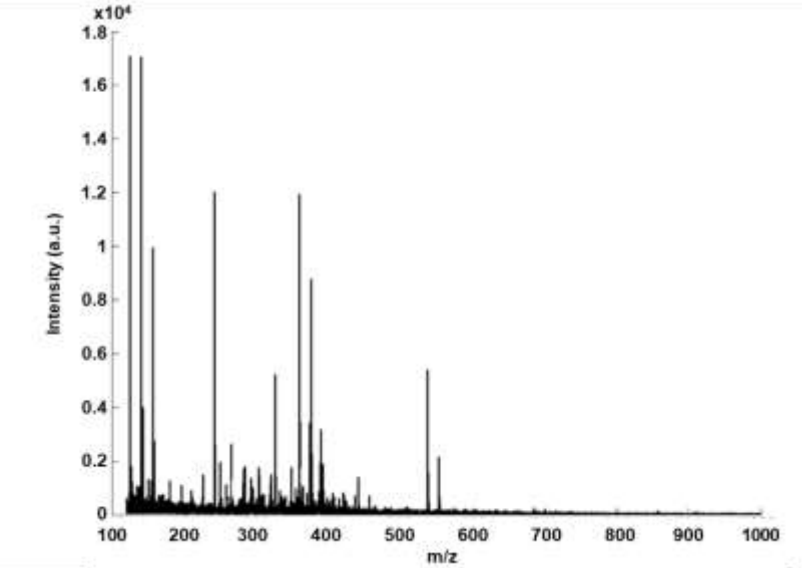

Figure S12. Mass spectrum from the MALDI-TOF MS of graphite flakes. No C_{60} is detected in this experiment and peaks fall at m/z values different to those of significant PAH oligomers.

S6. Correlation between the lifetimes of PAHs during e-beam and UV irradiation

As TEM and MALDI-TOF MS are utilised as sources of energy to induce transformations within PAH samples, careful comparison of the two sources and the energetics of the processes that were found to occur is essential.

C-H bond dissociation is considered to be a critical step in the transformations of PAHs, in both the photo-activated processes (MALDI-TOF MS measurements and in astrophysical environments) and e-beam induced processes (TEM imaging). The dissociation of this bond leads to reactive

intermediates – aryl radicals – responsible for the oligomerisation of PAHs and the formation of fullerenes. It is important to appreciate the differences and similarities of the conditions that the molecules experience in MALDI-TOF MS and in TEM, and attempt to rationalise why they lead to similar chemical transformations. A key difference is that under the e-beam of TEM, C-H bond dissociation is directly triggered by the kinetic energy transferred from a fast electron to a hydrogen atom, Figure S13a-b, while under the light-activated conditions in MALDI-TOF MS the molecule absorbs the photon and C-H dissociation occurs as a result of the relaxation of the photo-excited state of a PAH, Figure S13c-d.

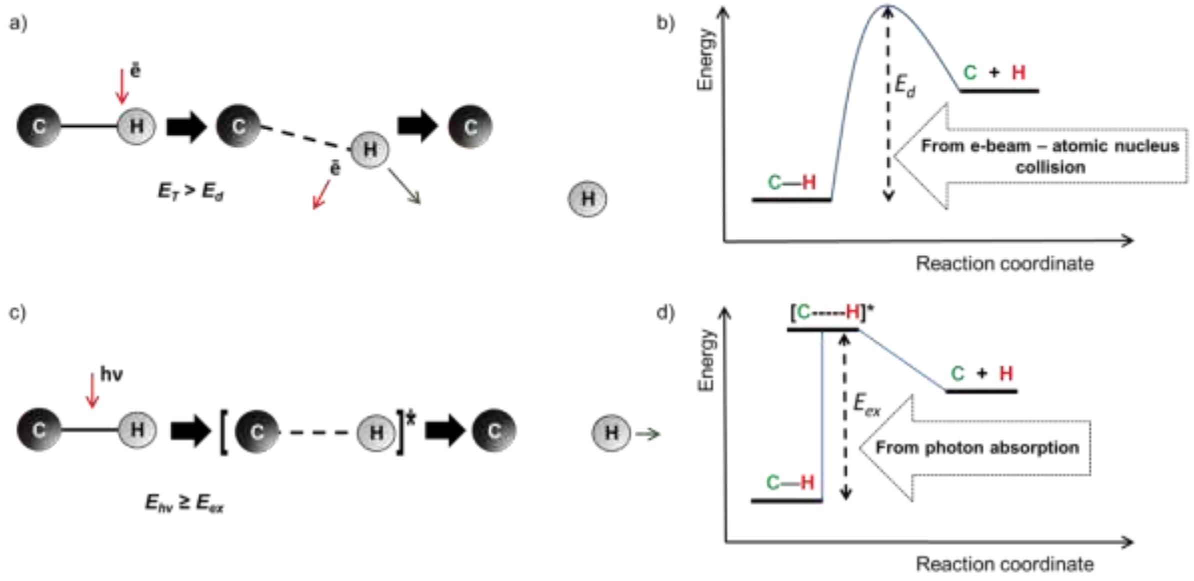

Figure S13. a) E-beam interaction with the atomic nucleus causes displacement of the atom from its equilibrium position and, if the transferred energy (E_T) is sufficiently high (i.e. $E_T > E_d$), dissociation of the chemical bond. b) Energy to promote a chemical reaction (energy threshold for decomposition, E_d) in TEM is supplied by collisions between the individual molecule and incident electrons. c) In contrast, in the light-activated process energy is supplied to the molecule by absorbed photons which, if high enough in energy ($E_{hv} \geq E_{ex}$), excite an electron in the molecule, generating an excited state which subsequently relaxes causing bond dissociation. d) Energy to promote the chemical reaction (energy threshold for excitation, E_{ex}) in light-activated conditions is therefore supplied by photons absorbed by the molecule.

The important steps required for the mechanisms proposed in this study are therefore likely to be important in the transformations seen in TEM also, and are the breaking of C-H bonds and C-C bonds, and the Stone-Wales rearrangement process. The bond enthalpies for C-H and C-C bonds, along with the activation energy of the Stone-Wales rearrangement are given in Table S3.

Although the kinetic energy of the electrons in the e-beam used in these experiments is 100 keV, not all of this energy is transferred to an atom in a collision. The maximum amount of transferrable energy (T_{max}) depends on the element of interest, and can be calculated using the following equation:

$$T_{max} = \frac{2 \cdot M \cdot E\ (E + 2m_e \cdot c^2)}{(M + m_e)^2 \cdot c^2 + 2M \cdot E}$$

where M is the atomic weight of the element, E is the energy of incident electrons, m_e is the mass of an electron, and c is the speed of light. Notably, T_{max} is inversely proportional to the atomic weight of the element, so that lighter elements, under the same TEM conditions, receive significantly more energy from the e-beam than heavier elements. T_{max} values for both carbon and hydrogen – the elements of significance in this study – are given in Table S3.

The dose rate in TEM can be altered manually, and the 'low-dose' and 'high-dose' values – used for imaging without causing transformations and used for initiating transformations, respectively – are given in Table S3. These are not absolute values, but do give an approximate rate of electrons impacting per nm^2 of the sample per second. A corresponding 'dose rate' of photons per nm^2 per second was calculated, using the number of photons per pulse, the pulse energy and duration, and the area of irradiation, and is shown in Table S3.

Table S3. Significant values for the study of PAH interactions with e-beam and UV irradiation, including the energies of important chemical processes, energies supplied by the 100 keV electrons in TEM and single photons (355 nm) in MALDI-TOF MS, and the respective dose rates of TEM and MALDI-TOF MS. † Li et al., 2007.

	$kJ\ mol^{-1}$	eV
C-H bond enthalpy	400 – 500	4 – 5
C-C bond enthalpy	~ 720	~ 7.5
Stone-Wales activation energy†	~ 870	~ 9
T_{max} of e^- to carbon in TEM	1930	20
T_{max} of e^- to hydrogen in TEM	23060	239
hν energy in MALDI-TOF MS	336.7	3.5
Low-dose TEM dose rate	$10^6\ e^-\ nm^{-2}\ s^{-1}$	
High-dose TEM dose rate	$10^9\ e^-\ nm^{-2}\ s^{-1}$	
High laser fluence 'dose rate'	43797 $h\nu\ nm^{-2}\ s^{-1}$	

When considering the irradiation of molecules, the term cross-section (σ) is used to express the probability of a particular interaction occurring. In TEM, the interaction cross-section is the probability of an incident electron interacting in some way with an atom; of particular relevance to this study is the cross-section for H atom ejection, *i.e.* the probability that an H atom on the edge of a PAH will be knocked out by an electron. When irradiating with UV photons, the analogous term is the cross-section for photon absorption – the probability of a photon being absorbed by a molecule – which can be used, in conjunction with the quantum yield of decomposition of a given molecule, to obtain a value for the cross-section for decomposition upon photon absorption. For the transformations of interest in this study, the term 'decomposition' is taken to represent C-H bond dissociation. The cross-sections for each technique can in turn be used to work out lifetimes of the

molecules under each form of irradiation. This has been done, and the results are presented in Table S4. The steps taken to obtain the values given in Table S4 are outlined below.

For UV irradiation, the values of cross-section of photon absorption for each of the four PAH molecules discussed in this study were obtained from the French-Italian database,(Malloci et al., 2007) and were found to be 7.06, 27.8, 8.52 and 112.26 megabarn (Mb, c.f. 1 barn = 10^{-28} m^2) for anthracene, pyrene, perylene and coronene, respectively. These values were then used to obtain a cross-section of decomposition following photon absorption (σ_d) for each molecule by multiplying with the quantum yield of decomposition – a unitless value between 0 and 1 that describes the efficiency of decomposition of a molecule following the absorption of a photon. The quantum yield of decomposition of a molecule depends heavily on the environment of the molecule and as such it can be difficult to determine. For the purpose of these calculations, the quantum yield of decomposition was assumed to be 1, *i.e.* the molecule decomposes upon absorption of the first photon, to act as an upper limit in our system.

$$\sigma_d = cross - section\ of\ photon\ absorption\ \times quantum\ yield\ of\ decomposition\ (taken\ as\ 1)$$

It should be noted that the term 'decomposition' could apply to a number of processes that result in the breaking down of the molecule. For this study, the loss of an H atom, *i.e.* C-H bond dissociation, is the first stage of the process that leads to the formation of oligomers and, in some cases, fullerenes. As these species are detected in MS, it is clear that to some degree this is the 'decomposition' that is occurring, and hence the values of lifetime under UV irradiation are taken to be the time it takes for a PAH molecule to undergo a transformation, most likely *via* the route of H loss.

To compare the cross-section values for each molecule with each other, they must be normalised to take into account the area of the molecule (calculated using the crystallographic dimensions of the different PAHs). This was achieved by multiplying σ_d for each molecule by its area (in barn), to obtain a unitless normalised cross-section (σ):

$$\sigma = \sigma_d \times area\ of\ molecule$$

To calculate the lifetimes of each of the PAHs under the conditions used, the 'dose rate' value for the UV laser in MALDI-TOF MS (given in Table S3 above) was converted to hv $barn^{-1}$ s^{-1}. The following equation was then used:

$$Lifetime\ (s) = \frac{1}{(\sigma_d(barn) \times dose\ rate\ (hv\ barn^{-1}s^{-1}))}$$

For e-beam irradiation, a similar process was carried out. The value of cross-section (σ_d), per atom, of H atom ejection from a coronene molecule was taken from Chamberlain *et al.*, 2015 to be 71.8 barn, and applied to all PAHs. This was then multiplied by the number of H atoms in each PAH, to obtain σ_d per molecule.

$$\sigma_d = \sigma_d(per\ atom) \times numberof\ H\ atoms$$

As before, a unitless normalised cross-section (σ) value for each molecule was obtained by multiplying σ_d by the area of the molecule, in barn. To calculate the lifetime of each PAH under the e-beam, the dose rate of the e-beam (Table S3) was converted to e^- $barn^{-1}$ s^{-1}, and the equation for lifetime shown previously was applied. The values obtained as a result of all of these calculations are presented in Table S4.

It is important to note that some of the values given in Table S4 can only be considered as estimates to allow comparison between the different PAHs. As mentioned previously, the calculations involving UV irradiation begin by assuming the quantum yield for decomposition to be 1. This provides an upper limit for the damage caused when a photon is absorbed, and hence the resultant lifetimes will be a lower limit, *i.e.* the shortest amount of time a PAH will survive under UV irradiation for before decomposition.

Similarly, the σ_d value for the ejection of an H atom from a PAH in e-beam irradiation that was used in these calculations was originally calculated for coronene molecules standing 'edge-on' (*i.e.* parallel) to the e-beam.(5) In the experiments described in this study, the PAH molecules are deposited on graphite flakes and are expected to be lying flat on the surface, *i.e.* perpendicular to the e-beam. The cross-section of H atom ejection in TEM has some dependence on the threshold energy – that is, the energy that the e-beam needs to transfer for atom ejection to occur. The threshold energy for molecules perpendicular to the e-beam will be slightly higher than for those positioned parallel to the beam, and hence the value of the cross-section (σ_d) is likely to be lower than that used in these calculations. In addition, the threshold energy will be marginally different for each PAH molecule, resulting in different cross-section values. However, differences in the threshold energies, and cross-sections, are larger between different elements than between the same element in different environments, *i.e.* on the edges of different PAHs. Finally, the calculated σ_d value that was used does not take into account inelastic interactions of electrons with the sample, and the cross-section therefore acts as a lower limit, indicating that the calculated lifetimes are an upper limit – the longest amount of time a molecule will survive under the e-beam before decomposition *via* C-H bond dissociation. Despite the approximate nature of this comparative analysis and the significant uncertainty of some experimental parameters, the expected timescales of photon and e-beam activated transformations in PAHs under our experimental conditions are remarkably similar (e.g. the lifetimes of PAHs molecules are within an order of magnitude, Table S4). This supports the methodological validity of the combined TEM and MALDI-TOF MS experimental approach developed for studying transformations of PAHs.

Table S4. Values of cross-section for decomposition (*i.e.* ejection of an H atom) and lifetimes of each PAH under the UV irradiation in MALDI-TOF MS and e-beam irradiation in TEM.

UV irradiation	Area of molecule		Cross-section (σ_d)*	Normalised cross-section (σ)	Dose rate	Lifetime
PAH molecule	Å^2	Barn	barn		hν barn^{-1} s^{-1}	s
Anthracene	42.81	4.281×10^9	7.06×10^6	0.001649	4.3797×10^{-6}	0.03234
Pyrene	53.44	5.344×10^9	2.78×10^7	0.005202	4.3797×10^{-6}	0.00822
Perylene	58.62	5.862×10^9	8.52×10^6	0.001453	4.3797×10^{-6}	0.02681
Coronene	80.15	8.015×10^9	1.12×10^8	0.014006	4.3797×10^{-6}	0.00204

e-beam irradiation	Area of molecule		Cross-section (σ_d)†	Normalised cross-section (σ)	Dose rate	Lifetime
PAH molecule	Å^2	Barn	barn		e$^-$ barn^{-1} s^{-1}	s
Anthracene	42.81	4.281×10^9	718.0	1.677×10^{-7}	0.1	0.01393
Pyrene	53.44	5.344×10^9	718.0	1.344×10^{-7}	0.1	0.01393
Perylene	58.62	5.862×10^9	861.6	1.470×10^{-7}	0.1	0.01161
Coronene	80.15	8.015×10^9	861.6	1.075×10^{-7}	0.1	0.01161

* σ_d = cross-section of decomposition following photon absorption

† σ_d = cross-section of H atom ejection

S7. Proposed mechanism for fullerene formation

In accordance with Figure 3 of the main article, and corresponding discussion on mechanistic details, Figure S14 details the mechanism proposed to form C_{60} from coronene. The mass spectrum given in Figure S14 shows a peak at m/z = 840 which is assigned to the fullerene C_{70}. The detection of this molecule suggests that the overall mechanism involves the formation of C_{70} initially, followed by its 'shrinking' via the loss of C_2 fragments to form C_{60}. This is supported by peaks in the mass spectrum indicating the detection of intermediate fullerenes: C_{68}, C_{66}, C_{64} and C_{62}. In a similar way to Figure 3 of the main article, peaks are seen in the mass spectrum of Figure S6 that are each separated by two mass units. One difference is that the coronene trimer structure, m/z = 888, contains no 'fjord' hydrogen sites. The initial steps of the mechanism in Figure S14, therefore, involve the loss of hydrogen atoms from the bay positions of the trimer, followed by C-C bond formation and Stone-Wales rearrangement; this is demonstrated by structures **10**, **11** and **12**. Following on from structure **12**, the next prominent MS peak falls at m/z = 856, which corresponds to a structure that would require the loss of C_2H_2. It is proposed that if the dissociation of a C_2H_2 fragment were to occur in the position indicated in structure **13**, it would result in a pseudo-bay area in which C-C bond formation would result in the creation of a four-membered ring that could be stabilised through Stone-Wales rearrangement at the indicated position. The result of this sequence is structure **14**, which is a candidate isomer for the mass spectrum peak seen at m/z = 856. Between structures **14** and **15** are three potential Stone-Wales rearrangements that would result in a molecule containing only five- and six-membered rings. In order to achieve the C_{70} fullerene structure, H atom loss, C-C bond formation and bond rearrangements are required. One example step is shown in Figure S14 from structure **15** to **16**, in which H-loss and C-C bond formation result in the formation of three new six-membered rings. It is expected that, driven by the formation of a low-energy fullerene molecule, structure **16** or similar will then continue to close up to form C_{70}, which will go on to form C_{60}.

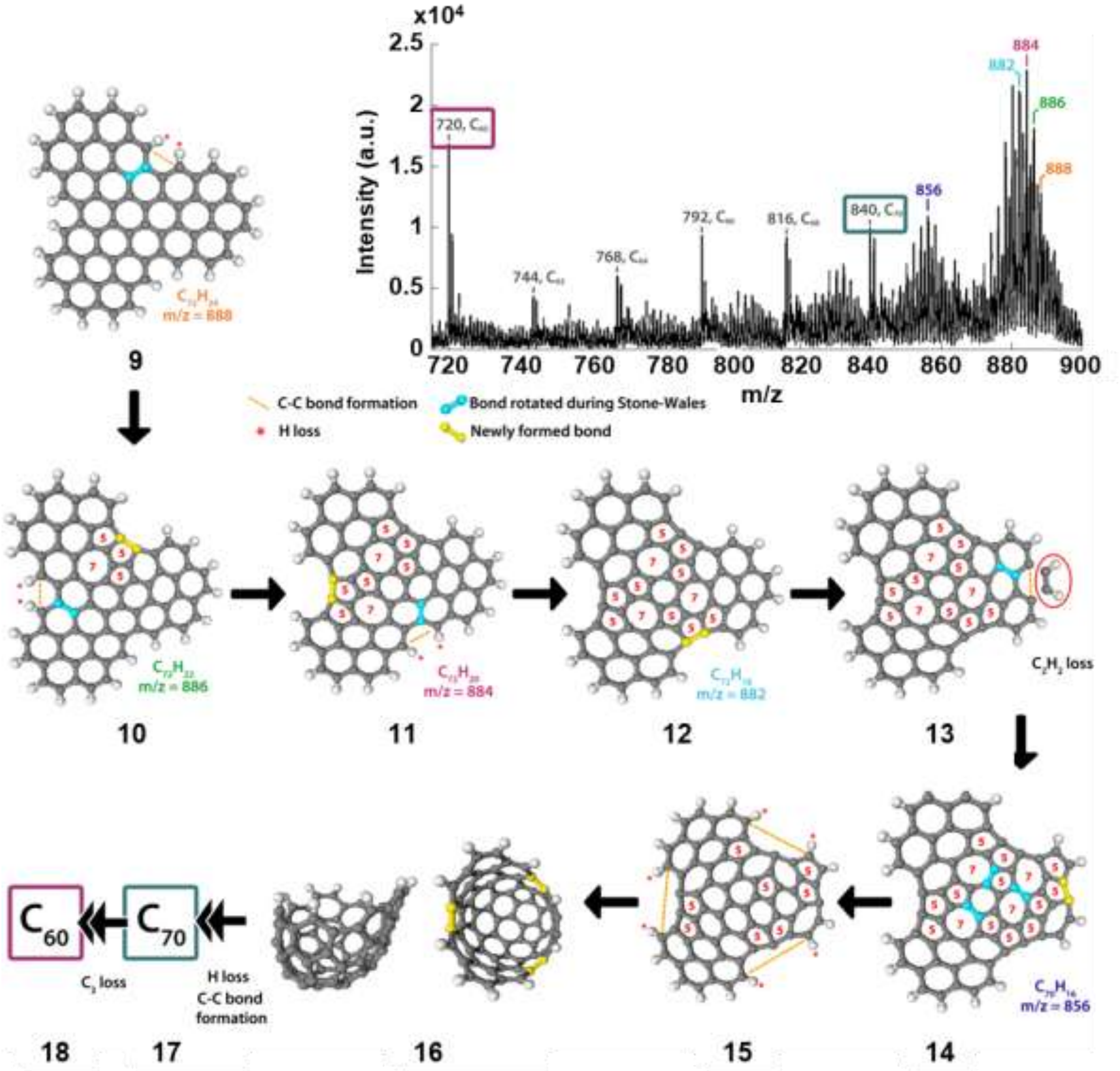

Figure S14. Section of the mass spectrum resulting from UV irradiation of coronene (top) and suggested mechanistic steps showing the transformation from the coronene trimer to C_{60}.

References

Chamberlain, T. W., Biskupek, J., Skowron, S. T., Bayliss, P. A., Bichoutskaia, E., Kaiser, U., and Khlobystov, A. N., (2015). Isotope Substitution Extends the Lifetime of Organic Molecules in Transmission Electron Microscopy. *Small* 11, 622-629. doi.org/10.1002/smll.201402081

Chuvilin, A., Kaiser, U., Bichoutskaia, E., Besley, N. A., and Khlobystov, A.N., (2010). Direct transformation of graphene to fullerene. *Nat. Chem.* 2, 450–453. doi.org/10.1038/nchem.644

Li L., Reich, S., and Robertson, J., (2005). Defect energies of graphite: Density-functional calculations. *Phys. Rev. B* 72, 184109. doi.org/10.1103/PhysRevB.72.184109

Malloci, G., Joblin, C., and Mulas, G., (2007). On-line database of the spectral properties of polycyclic aromatic hydrocarbons. *Chem. Phys.* 332, 353-359. doi.org/10.1016/j.chemphys.2007.01.001

Santana, A., Zobelli, A., Kotakosk,i J., Chuvilin, A., and Bichoutskaia, E., (2013). Inclusion of radiation damage dynamics in high-resolution transmission electron microscopy image simulations: The example of graphene. *Phys, Rev, B* 87, 094110-1-094110-8. doi.org/10.1103/PhysRevB.87.094110
